# Lipid Composition Is Critical for Accurate Membrane Permeability Prediction of Cyclic Peptides by Molecular Dynamics Simulations

**DOI:** 10.1021/acs.jcim.2c00931

**Published:** 2022-09-02

**Authors:** Masatake Sugita, Takuya Fujie, Keisuke Yanagisawa, Masahito Ohue, Yutaka Akiyama

**Affiliations:** † Department of Computer Science, School of Computing, Tokyo Institute of Technology, W8-76, 2-12-1 Ookayama, Meguro-ku, Tokyo 152-8550, Japan; ‡ Middle-Molecule IT-Based Drug Discovery Laboratory (MIDL), Tokyo Institute of Technology, W8-76, 2-12-1 Ookayama, Meguro-ku, Tokyo 152-8550, Japan

## Abstract

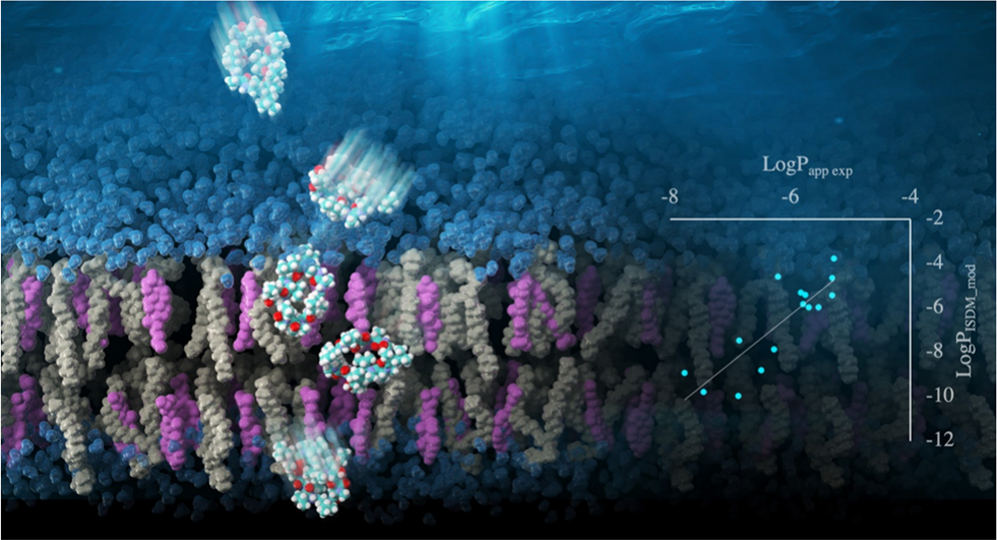

Cyclic peptides have attracted attention as a promising pharmaceutical modality due to their potential to selectively inhibit previously undruggable targets, such as intracellular protein–protein interactions. Poor membrane permeability is the biggest bottleneck hindering successful drug discovery based on cyclic peptides. Therefore, the development of computational methods that can predict membrane permeability and support elucidation of the membrane permeation mechanism of drug candidate peptides is much sought after. In this study, we developed a protocol to simulate the behavior in membrane permeation steps and estimate the membrane permeability of large cyclic peptides with more than or equal to 10 residues. This protocol requires the use of a more realistic membrane model than a single-lipid phospholipid bilayer. To select a membrane model, we first analyzed the effect of cholesterol concentration in the model membrane on the potential of mean force and hydrogen bonding networks along the direction perpendicular to the membrane surface as predicted by molecular dynamics simulations using cyclosporine A. These results suggest that a membrane model with 40 or 50 mol % cholesterol was suitable for predicting the permeation process. Subsequently, two types of membrane models containing 1-palmitoyl-2-oleoyl-*sn*-glycero-3-phosphocholine and 40 and 50 mol % cholesterol were used. To validate the efficiency of our protocol, the membrane permeability of 18 ten-residue peptides was predicted. Correlation coefficients of *R* > 0.8 between the experimental and calculated permeability values were obtained with both model membranes. The results of this study demonstrate that the lipid membrane is not just a medium but also among the main factors determining the membrane permeability of molecules. The computational protocol proposed in this study and the findings obtained on the effect of membrane model composition will contribute to building a schematic view of the membrane permeation process. Furthermore, the results of this study will eventually aid the elucidation of design rules for peptide drugs with high membrane permeability.

## Introduction

Cyclic peptides have attracted the attention of many pharmaceutical companies worldwide as a new modality due to their potential to permeate the plasma membrane and interact with their targets with antibody-like high specificity, even for flat protein surfaces.^1−3^ The ability of cyclic peptides may enable access to previously undruggable targets, such as intracellular protein–protein interactions (PPIs), with fewer side effects. The availability of efficient synthetic and screening systems^4,5^ is an outstanding feature of cyclic peptides compared to other macrocyclic and “beyond the rule of 5” compounds.^6,7^ However, cyclic peptides typically exhibit poor membrane permeability. Establishing rules for designing peptides with high membrane permeability is essential to succeed in cyclic peptide drug discovery. From this point of view, the membrane permeation mechanism of peptides, especially permeation by passive diffusion, has been actively studied.

Membrane permeation of molecules by passive diffusion occurs in all cell types. Furthermore, most drug molecules are transported across the membrane by passive diffusion. Therefore, the membrane permeation process by passive diffusion of molecules has been widely studied, mainly targeting small molecules. The membrane permeability of many molecules has been determined by various experimental techniques.^8−12^ These experimental data revealed that the partition coefficients of small molecules between water and organic solvents and their solubility in water are strongly related to membrane permeability. It has been suggested that molecular weight also has a significant effect on membrane permeability.^13,14^ In addition, mechanisms have been investigated through molecular simulations.^10,11,15−20^ It is known that rough trends in membrane permeability of small molecules can be revealed by the classical homogeneous solubility–diffusion model^21−23^ or its modified versions, i.e., a three slab model,^24^ an inhomogeneous solubility–diffusion model (ISDM),^15^ and its extension to multidimensional reaction coordinates.^19^ These models suggest that the affinity of the molecule to the membrane relative to that in aqueous solution and the diffusion rate in the membrane determine the membrane permeability. However, it has also been proposed that the behavior in the solution, such as the diffusion rate at the unstirred water layer (UWL), has a significant impact on the membrane permeability. The data indicate that the solubility–diffusion model alone is unable to completely predict the membrane permeability.^25,26^

Compared to conventional small molecules, cyclic peptides have large molecular weights and flexibility. Therefore, the membrane permeation process of cyclic peptides is considered to be more complex than that of small molecules, especially for the large cyclic peptides that exceed 10 residues, which are prone to achieve high affinity with the target. Many experiments have suggested that peptides that can hide polar groups in the membrane, by forming intramolecular hydrogen bonds, can have higher membrane permeability.^27−33^ These findings have been supported by simulation data in bulk water and organic solvents.^34−37^ However, it is still difficult to accurately predict the membrane permeability of cyclic peptides by computational techniques.

**Figure 1:**
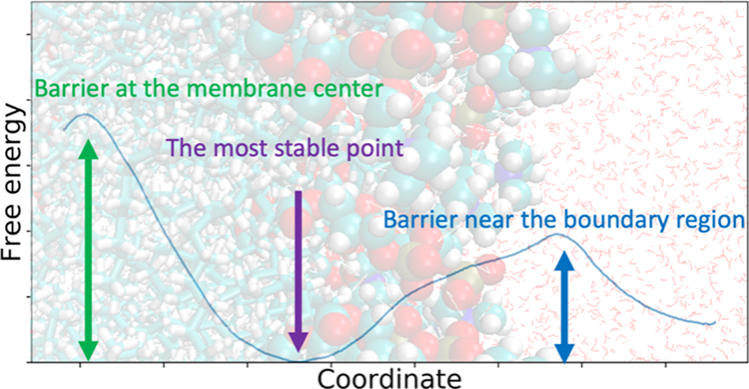
Typical example of the potential of mean force (PMF) of a cyclic peptide permeating a POPC membrane, revealed in a previous study.^39^

Due to their large molecular weight and flexibility, few reports have been published on simulation studies of membrane permeation processes through the lipid bilayer.^38^ However, we recently reported the prediction results of membrane permeability for more than 100 six- and eight-residue peptides through 1-palmitoyl-2-oleoyl-*sn*-glycero-3-phosphocholine (POPC) membranes based on molecular dynamics (MD) simulations with inhomogeneous solubility–diffusion model (ISDM).^39^ The results showed that a high free energy barrier was observed at the center of the membrane. Furthermore, another free energy barrier and the most stable point were observed near the boundary region between the membrane and water (Figure 1), providing information that cannot be obtained from simulations using bulk solution models. These findings suggest that the lipid membrane is not just a surrounding medium but also an active player in determining membrane permeability.

Considering that the lipid membrane is not just a surrounding medium, it is necessary to explore the relationship between the components of lipid membranes and permeability. Usually, a homogeneous lipid bilayer, made from POPC,^11,17−19,38,40^ 1,2-dioleoyl-*sn*-glycero-3-phosphocholine (DOPC),^18,20,41,42^ or occasionally 1,2-dipalmitoyl-*sn*-glycero-3-phosphocholine (DPPC)^15,18,43^ for example, is often used as a model membrane in MD simulations. However, in living cells, the concentration of cholesterol in the plasma membrane can reach approximately 50 mol % in some cases,^44^ and it is assumed that the membranes are stiffer than homogeneous membranes containing unsaturated fatty acids.^45^ Indeed, it has been shown experimentally^46^ and theoretically^47,48^ that the permeability of small molecules depends on the concentration of cholesterol in the membrane. In particular, it has been shown that cholesterol has an inhibitory effect on membrane permeation of a small molecule by reducing the partition of molecules into the membrane.^48,49^ It was attributed to the obstruction of penetration of water molecules and lipid heads into the inner part of the membrane. However, it has not been clarified how the presence or absence of cholesterol affects the membrane permeation process of cyclic peptides.

In the present study, we focused on the effect of cholesterol concentration in lipid membranes and performed two calculations. First, the behavior of cyclosporine A (CSA) in lipid bilayers with cholesterol concentrations ranging from 0 to 50 mol % in 10 mol % intervals was analyzed, and the membrane permeability at each cholesterol concentration was predicted. The analyzed data of CSA suggest that a membrane model with 40 or 50 mol % cholesterol was suitable for permeability prediction. Next, we predicted the membrane permeability of 18 ten-residue peptides using lipid bilayers with a cholesterol concentration of 40 and 50 mol %. The permeability of peptides was experimentally elucidated by Furukawa et al., we call them Furukawa data.^32^ The Furukawa data were chosen as the target of this study because they represented a unique data set showing many experimental values of membrane permeability coefficients for peptides with 10 or more residues, which were almost nonexistent at the beginning of this project. For both membrane models, we obtained predicted membrane permeability coefficients which have correlation coefficients of *R* > 0.8 with the experimental values.

## Results and Discussion

### Analysis of Potential of Mean Force, Number of Hydrogen Bonds, and Membrane Permeability of Cyclosporine A at Various Cholesterol Concentrations

We first analyzed the differences in a schematic view of the membrane permeation process of CSA at different cholesterol concentrations based on MD simulations using replica exchange with solute tempering (ST) and replica-exchange umbrella sampling (REST/REUS) methods.^50^ The key point of this protocol is that the maximum temperature of the solute molecule is set significantly high to 980 K at which the cis–trans isomerization of the ω angle of the peptide bonds on N-substituted peptides and proline can be explored, as shown in Figure S1b. Consequently, the conformation of the peptide can be efficiently explored. The details of the protocol are given in the Methods section.

**Figure 2:**
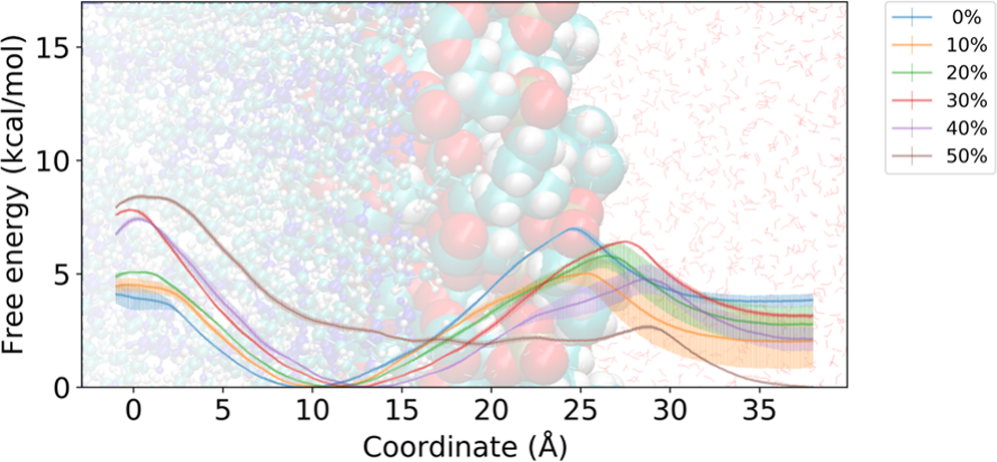
PMFs of the CSA against the reaction coordinate *z*. The legend shows the colors associated with the mole fraction of cholesterol in the model membrane. The rough positions of lipid and solvent molecules are depicted in the background. Solvent molecules are drawn as line representations. The lipid molecules are drawn as a ball-and-stick model, but only the phosphatidylcholine moiety is drawn as a space-filling model. The cholesterol molecules are colored blue. The error bars are determined as the standard deviation of PMFs calculated independently from the trajectories of the first and second 150 ns.

The predicted potential of mean force (PMF) along the reaction coordinate *z* is shown in Figure 2. The reaction coordinate *z* is defined as the distance on the axis orthogonal to the membrane plane between the center of mass of all nitrogen atoms of phosphatidylcholine and that of nitrogen atoms consisting of the peptide bonds in the main chain. To show the degree of convergence, the PMFs calculated from the trajectories of the first 150 ns and second 150 ns and the mean absolute error (MAE) between the two PMFs are shown in Figure S2 and Table S1.

The center of the membrane corresponds to *z* = 0 Å and the free energy barrier around there increases as the cholesterol concentration increases. By contrast, the free energy difference between the PMF minimum near *z* = 12 Å and the peak near *z* = 25 Å decreased. Comparing the difference in free energy between the PMF minima near *z* = 12 and 37.5 Å, it was revealed that the inside of the membrane was destabilized as the concentration of cholesterol increased. Interestingly, as cholesterol concentration increased from 40 to 50 mol %, the free energy minimum near *z* = 12 Å disappears.

Most importantly, the free energy barrier around *z* = 0 Å is too low compared to the barrier around *z* = 25 Å for a pure POPC membrane without cholesterol, despite being commonly used in simulations. This is inconsistent with the experimental findings. The Madin–Darby canine kidney cell (MDCK) assay data for CSA and its derivatives conducted by Naylor et al.^28^ showed that peptides with AlogP lower than 4 had a tendency for membrane permeability to increase in proportion to AlogP. AlogP is a predicted value of the water/octanol partition coefficient.^51^ These results indicate that the permeability of peptides with AlogP < 4 is governed by the process of crossing the free energy barrier around *z* = 0 Å, which is mainly caused by the penalty of dehydration.^39^ Although other factors may affect the membrane permeability of CSA, where AlogP is slightly greater than 4, it is assumed that the free energy barrier near *z* = 0 Å is one of the major factors determining membrane permeability. However, the simulation results for CSA on cholesterol-free POPC membranes indicate that CSA is more stable inside the membrane than outside and that the free energy barrier at the center of the membrane has little effect on membrane permeability. The results for systems with cholesterol ratios greater than 30 mol % are consistent with the experimental findings, as the barrier near *z* = 0 Å is the rate-limiting factor. In addition, predicted membrane permeability based on the data obtained from the simulation using a membrane with 50 mol % cholesterol is closest to the experimental value,^28^ as shown in Table 1. These results suggest that a membrane consisting of 50 mol % cholesterol seems suitable for predicting PMF relating to the membrane permeation process of the CSA.

**Table 1:** Log Scaled Experimental^28^ and Predicted Membrane Permeability Values, with Units of cm/s, to the Base 10^a^

exp.	0%	10%	20%	30%	40%	50%
–5.85 ± 0.06	–3.98 ± 0.07	–3.01 ± 0.98	–3.45 ± 0.17	–4.37 ± 0.05	–4.17 ± 0.08	–5.23 ± 0.39

a Percentage indicates the mole fraction of cholesterol on the model membrane. Errors for predicted values were estimated as the standard deviation of the permeability calculated from the first and second 150 ns MD trajectories.

Regarding the data at 10 mol % cholesterol concentration, which show that PMF does not converge well, we investigated the reason for the poor PMF convergence. For this purpose, several analyses were performed for the entire trajectory, including 300 ns of the equilibration process and 300 ns of the production run. In particular, we focused on the region *z* = 25–27 Å due to the large deviation of the PMF in this region. First, the angle between the principal axis of inertia corresponding to the maximum eigenvalue (hereafter referred to as the short axis) and the reaction coordinate *z* at *z* = 27 Å was plotted versus time in Figure S3. The plot and error bars show the mean value every 25 ns and the standard deviation over that interval. The short axis represents the axis perpendicular to the plane when the peptide is considered as a flat disk. This plot shows that the angle between the short axis and the *z*-coordinate increases gradually with the simulation time, indicating an increase of structures in which the peptide is anchored to the membrane, as shown in Figure S4. This can also be implied from the plots of area per lipid (APL) versus time at four locations shown in Figure S5; at the membrane center (*z* = 0 Å), near the free energy minima (*z* = 10.5 Å), near the lipid headgroup (*z* = 27 Å), and outside the membrane (*z* = 37.5 Å). When the peptide is at *z* = 0 and 10.5 Å, the profile oscillates at an average value of around 62 Å^2^ and no drift is observed. On the other hand, when the peptide is at *z* = 37.5 Å, it oscillates at a slightly lower value of around 61 Å^2^. This is due to the fact that the plane size of the simulation box is smaller because the peptide is outside the membrane. For the data around *z* = 27 Å, the APL appears to drift slightly in the latter 150 ns of the production run. This may be due to an increase in the number of structures in which the peptide penetrates the membrane, causing a slight increase in the APL.

The diffusion coefficient profiles are also shown in Figure S6. From the figure, it can be seen that the diffusion coefficient values in the membrane are almost unaffected by the cholesterol concentration. The lack of influence of cholesterol on the diffusion coefficient along the direction perpendicular to the membrane surface has also been reported elsewhere.^49^ On the other hand, the profile on the outside of the membrane changes depending on the concentration of cholesterol. This is due to the reduction in APL with increasing cholesterol concentration, as shown in Table 5, which in turn increases the membrane thickness. Although it may be safer to apply sampling to longer distances for CSA, we consider the predicted values of the membrane permeability coefficient are less affected since the PMF values appear to converge at *z* = 37.5 Å and the free energy minimum is located between *z* = 10 and 15 Å except for the 50 mol % cholesterol data.

**Figure 3:**
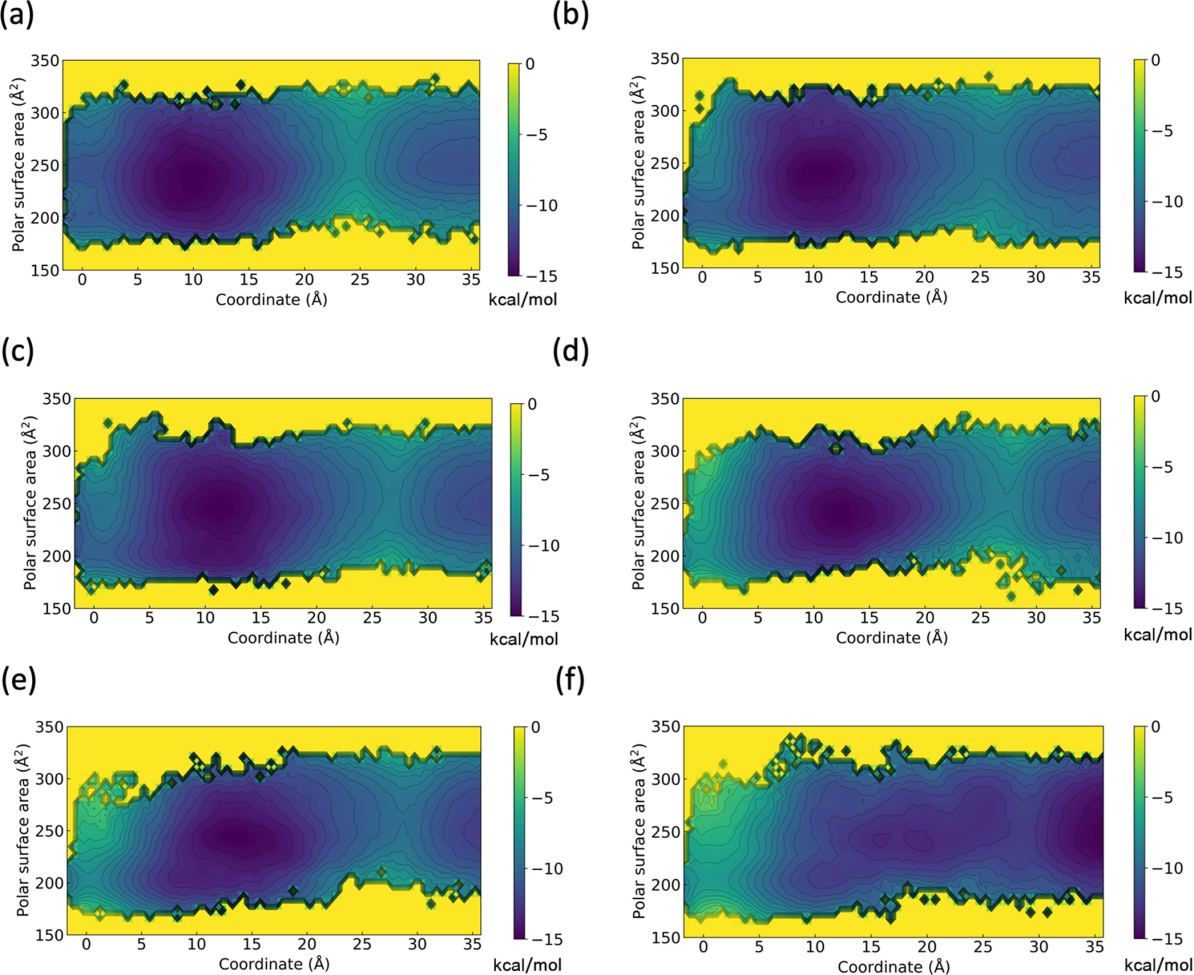
2D-PMF of CSA across model membrane with (a) 0 mol %, (b) 10 mol %, (c) 20 mol %, (d) 30 mol %, (e) 40 mol %, and (f) 50 mol % fraction of cholesterol along the two reaction coordinates. The reaction coordinate *z* and polar surface area were used as the horizontal and vertical axes, respectively.

To clarify the effect of the concentration of cholesterol on the conformation of the peptide throughout the membrane permeation process, the two-dimensional (2D) PMFs for the polar surface area (PSA) and reaction coordinate *z* were calculated and are shown in Figure 3. As for the membrane permeation process, there seems to be no major difference in the distribution of conformations from the outside of the membrane to the region around *z* = 10–15 Å, despite the difference in stability between the outside and inside of the membrane, as seen in one-dimensional (1D) PMFs. There was almost no bias in the polar surface area outside the membrane, resulting in a variety of conformations. Even in a relatively stable region of PMF near *z* = 10–15 Å, there were a variety of conformations. However, a large difference in the distribution of conformations near the center of the membrane was observed depending on the cholesterol concentration. In other words, for the 0 mol % cholesterol fraction, conformations with a relatively large polar surface area of PSA = 250 Å^2^ in addition to the conformations of PSA = 200 Å^2^ were shown to be stable near the membrane center. However, the conformations with a relatively large polar surface area disappear as the membrane cholesterol concentration increases.

To show a more detailed aspect of the conformations of the CSA at each position of the membrane with different cholesterol mol %, trajectories were projected onto eigenvectors obtained from principal component analysis of the dihedral angles of the main chain (φ, ψ, ω).^52^ PMFs against the first and second principal axes are shown in Figures S7–S11. The PMF calculated based on the projected trajectories at *z* = 0–5 and 30–37.5 Å obtained from the simulation with a cholesterol-free membrane is shown in Figure S7. Representative snapshots, average polar surface area, average hydrogen-bond number, and the average number of peptide bonds with a cis-type ω dihedral angle around the free energy minima are also shown. The conformations included in the region of the first principal component values of 2–3 correspond to the closed structures that are commonly found in organic solvents.^53,54^Figure S7 shows that the structure at PSA = 200 Å^2^ is a closed structure, whereas the structure at PSA = 250 Å^2^ is a mixture of several open structures. Figures S8–S11 show the PMFs calculated based on the projected trajectories of various model membranes with different cholesterol fractions at *z* = 30–37.5, 9–10.5, 4–5, and 0–1 Å, respectively. Similar profiles representing various conformations were observed outside the membrane and around *z* = 10 Å regardless of the membrane composition used in the calculation, as shown in Figures S8 and S9. In contrast, the stability of the closed conformations near the center of the membrane differed depending on the cholesterol concentration of the membrane, as shown in Figures S10 and S11. The dependency of the content of closed conformations on the cholesterol concentration of the membrane at several positions of the membrane is shown in Figure S12. The figure shows a more distinct difference. In the system with 0 mol % cholesterol content, there is a little closed structure even at the center of the membrane. In the system with 10–30 mol % cholesterol content, the closed structure at the center of the membrane is dominant, but when the peptide was moved away from the center to *z* = 5 Å, the fraction of the closed structure decreases to approximately 40%. When the cholesterol concentration exceeded 40%, the closed conformation becomes dominant even at *z* = 5 Å, and the closed structure remains stable up to *z* = 9 Å away from the center of the membrane.

CSA adopts a “closed” conformation in the crystal structure and in nonpolar solutions, where all hydrogen-bond donors form intramolecular hydrogen bonds.^53,54^ Although the extent to which CSA has a closed structure near the center of real cell membranes is unknown, many studies have shown a relationship between the closed structure of peptides in organic solvents and membrane permeability,^27,33,36,37,55,56^ suggesting that a certain percentage of closed structures is present in cell membranes. Notably, the ratio of closed structures at the center of the membrane in the pure POPC membrane model was very small (20%). Therefore, a membrane model with ≥10 mol % cholesterol, which reproduces a closed CSA conformation of >60% in the membrane, is considered reasonable to simulate the membrane permeation process.

**Figure 4:**
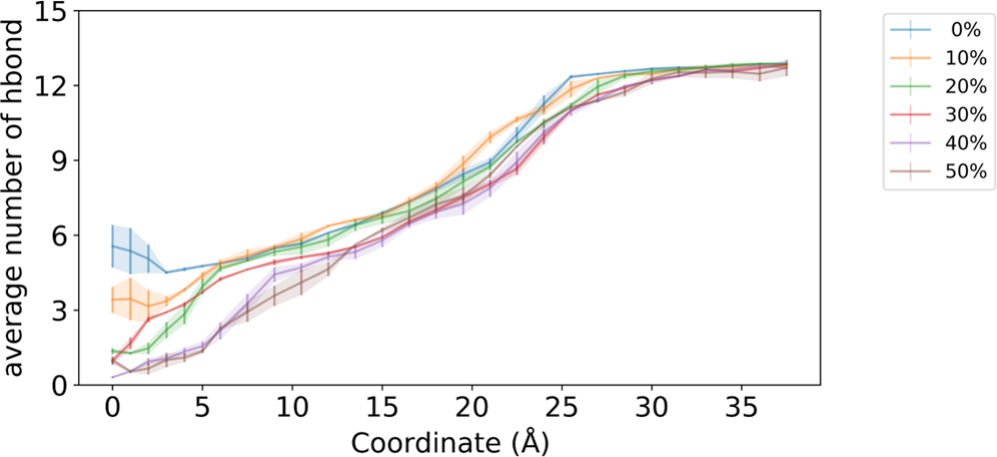
Average number of hydrogen bonds between peptide and water molecules against reaction coordinate *z*. The legend shows the colors associated with the mole fraction of the cholesterol in the model membrane. The error bars are determined as the standard deviation of profiles calculated independently from the trajectories of the first and second 150 ns.

The results of the 2D-PMF-based analysis of PSA and *z*-coordinate, showed that there is a difference in the distribution of the peptide conformations near the center of the membrane depending on the membrane cholesterol concentration. In the analysis of intermolecular interactions, we found that the change in the percentage of closed structures near the center of the membrane, associated with these differences in cholesterol concentration, was dependent on the ability of water to penetrate the membrane. The average number of hydrogen bonds between the peptide and water is plotted against *z* in Figure 4. This profile shows that the peptide detaches from the water molecules as it moves toward the center of the membrane, and a large difference in the profile in the region of *z* < 5 Å was observed, depending on the composition of the membrane. In membranes with cholesterol concentration of 40 or 50 mol %, the peptides were almost completely dehydrated in this region. Meanwhile, in membrane models with a cholesterol concentration of 10 mol % or less more than three hydrogen bonds between water and peptides were maintained on average. Interestingly, the simulation results for the cholesterol-free POPC membrane showed that the interaction with water increased from *z* = 5 Å towards the center of the membrane. A representative snapshot obtained from the simulation at *z* = 0 Å using a cholesterol-free membrane model is shown in Figure S13. As discussed previously, many studies have shown a relationship between the closed structure of peptides in organic solvents and membrane permeability, and such an abundance of water molecules in the membrane does not appear realistic. Therefore, a membrane with a cholesterol concentration of 40 or 50 mol % would be more appropriate for predicting the membrane permeation process since the peptide is almost completely dehydrated near the center of the membrane.

### Membrane Permeability Prediction for a Variety of Peptides at 40 and 50 mol % Cholesterol Concentration

From the viewpoint of whether the rate-limiting step of the permeation process of CSA occurs at the center of the membrane, it was suggested that a membrane model with a cholesterol concentration of 30 mol % or higher would be appropriate. Based on the average number of hydrogen bonds between CSA and water molecules at the center of the membrane, a membrane model containing 40 mol % or more cholesterol appeared to be optimal for predicting membrane permeability. The predicted membrane permeability was closest to the experimental value when the membrane model used contained 50 mol % cholesterol, but the shape of PMF is different compared to others. It is unclear whether the differences between PMFs obtained from simulations using membranes with 50 mol % cholesterol concentration and others will have a positive or negative impact on the prediction of membrane permeability. These data suggest that the membrane model with 40 or 50 mol % cholesterol is appropriate for predicting the membrane permeation process and permeability. To confirm the efficacy of the membrane model with 40 and 50 mol % cholesterol in predicting membrane permeability, we predicted the membrane permeability of 10-residue peptides from the study published by Furukawa et al.^32^ using a membrane containing 40 and 50 mol % cholesterol. The experimental and AlogP values are listed in Table S2, and the chemical structures are shown in Figure 7.

**Figure 5:**
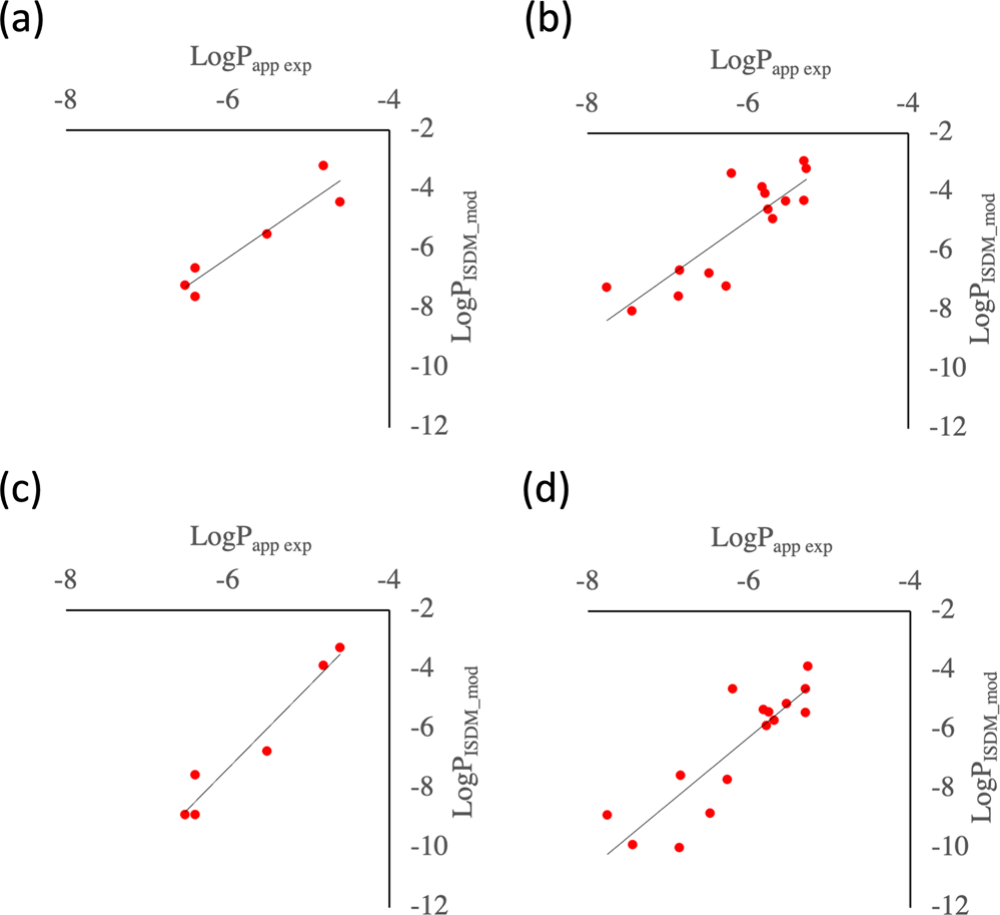
Scatter plot illustrating the experimental and calculated membrane permeability for the (a) Furukawa:MDCK and (b) Furukawa:PAMPA data using a membrane model containing 40 mol % cholesterol, and (c) Furukawa:MDCK and (d) Furukawa:PAMPA data using a membrane model containing 50 mol % cholesterol.

Scatter plots of the calculated membrane permeability, using a membrane model containing 40 mol % cholesterol, and experimental values are shown in Figure 5a,b. Figure 5a shows six peptides for which MDCK assay data exist, we termed them as Furukawa:MDCK data, and Figure 5b shows 15 peptides with AlogP < 4 from library B in the literature, which have PAMPA assay data, referred to as Furukawa:PAMPA data. The correlation coefficients between the experimental and calculated membrane permeabilities for Furukawa:MDCK data and Furukawa:PAMPA data were *R* = 0.94 and 0.85, respectively, qualitatively showing excellent agreement with the experimental data. Figure 5c,d shows scatter plots of the calculated membrane permeability for Furukawa:MDCK and Furukawa:PAMPA data, using a membrane model containing 50 mol % cholesterol, and experimental values. The correlation coefficients between the experimental and calculated membrane permeability for Furukawa:MDCK data and Furukawa:PAMPA data were *R* = 0.97 and 0.87, respectively, qualitatively showing excellent agreement with the experimental data. These results demonstrate the robustness of the present protocol, with either 40 or 50 mol % cholesterol-containing membranes, in terms of being able to make qualitative predictions of the membrane permeability of peptides other than CSA using a membrane model tuned with the use of CSA. The mean absolute errors (MAEs) between log scaled experimental and predicted membrane permeability values, with units of cm/s, to the base 10 are shown in Table 2. For the Furukawa:MDCK data, MAE is smaller when using a membrane model containing 40 mol % cholesterol. For the Furukawa:PAMPA data, MAE was smaller when using a membrane model containing 50 mol % cholesterol. The MAE between the PMFs estimated from the trajectories of the first 150 ns and the second 150 ns is shown in Tables S2 and S3 to indicate the degree of convergence of the simulation. PMFs and diffusion coefficients along the *z*-coordinate are shown in Figures S14–S25 as references. Notably, the correlation coefficient between the experimental and calculated values for peptides in the Furukawa:MDCK data using cholesterol-free membranes was *R* = −0.79, as shown in Figure S26, which does not represent a plausible result.

**Table 2:** Correlation Coefficients and MAE Values between Log Scaled Experimental and Predicted Membrane Permeability for the Furukawa Data^32^^,^^a^

data	cholesterol contents (mol %)	*R*	MAE
Furukawa:MDCK	40	0.94	0.66
Furukawa:MDCK	50	0.97	1.72
Furukawa:PAMPA	40	0.85	1.26
Furukawa:PAMPA	50	0.87	1.12

a MAE denotes the mean absolute error,  between experimental and calculated membrane permeability for target peptides.

Which cholesterol concentration is best suited for predicting membrane permeability? There is no clear answer to this question. This is because real cellular membranes are more complex, consisting of a much greater variety of lipid molecules and often glycosylated. Furthermore, their composition varies for each cell. In addition, the model membrane used in the PAMPA assay is composed of a significantly thicker membrane support (several hundred micrometers) compared to the cell membrane (several nanometers), filled with an organic solvent such as dodecane, and is very different from a lipid bilayer membrane. The order of magnitude of the permeability obtained may also vary depending on the solution conditions under which the experiment was performed. It would be ideal to perform simulations using a lipid membrane model with the same composition as the cells being targeted. However, such a calculation is nearly impossible with the resources available at this time. Instead, it would be useful to explore models that are qualitatively but broadly predictive of data from many cellular and PAMPA assays. The results of this study suggest that the POPC membrane model with 40 or 50 mol % cholesterol, in which the peptide is almost completely dehydrated near the center of the membrane, is a widely useful membrane model for predicting permeability.

Intriguingly, the work of Sugita et al.^39^ showed correlation coefficients of approximately *R* = 0.6 between the predicted and experimental values of the membrane permeability of six- and eight-residue peptides even though a cholesterol-free POPC membrane was used. Those results are in contrast to those in the present work. To clarify the reason for this discrepancy, the number of hydrogen bonds between the peptides and water is plotted in Figure S27 against the reaction coordinate *z* for the three peptides whose AlogP values are close to CSA, extracted from the simulation trajectory of six-residue peptides permeating the homogeneous POPC membrane obtained in the work of Sugita et al.^39^ The profile of hydrogen bonds between peptides and water is similar to that of the CSA data, as shown in Figure 4, in regions other than the center of the membrane, and it can be observed that the interaction with water decreases toward the center of the membrane. However, the average number of hydrogen bonds with water around *z* = 0 Å is approximately 2, a relatively small value compared to the cholesterol-free data for CSA. This may be due to the fact that the peptides used in that work^39^ were much smaller than CSA. Even though pure POPC membranes are softer than cholesterol-mixed membranes and allow water to penetrate the interior more easily, the probability of water reaching the center of the membrane, where the hydrophobicity is greatest, is very low. For relatively small cyclic peptides (≤8 residues), the edge of the peptide molecule was located far from the membrane surface when the center of mass of the peptide was at the center of the membrane, and the interactions with the water molecules were sufficiently reduced even when a pure POPC membrane was used. In contrast, for relatively large cyclic peptides (≥10 residues), as shown in Figure S13, even if the center of mass of the peptide molecule was located at the center of the membrane, the edge of the peptide molecule was close to the membrane surface, forming a route for water across the membrane along the hydrophilic groups and main chain of the peptide. Therefore, using a pure POPC membrane would retain a large number of interactions with water, even if the center of mass of the peptide was at the center of the membrane, resulting in unrealistic predictions. Because cholesterol-rich membranes have a dense structure, the water molecules are unlikely to penetrate through the peptide into the membrane,^48,49^ even if the edges of the peptide are easily accessible to water. Therefore, even a large peptide such as CSA is almost completely dehydrated at the center of the membrane. This indicates that our results are consistent with those of Sugita et al.^39^ and that large peptides are more sensitive to the composition of the membrane than small peptides.

## Conclusions

In this study, we analyzed the changes in PMF, the number of hydrogen bonds, and the PSA during the permeation process. Furthermore, we analyzed the effect of the membrane cholesterol concentration on the predicted membrane permeability, first targeting CSA. In the case of the commonly used cholesterol-free membranes, peptides are not completely dehydrated because water can easily access the membrane interior. Consequently, peptides can pass through the membrane without complete dehydration, resulting in very high and unrealistic membrane permeability predictions. On the other hand, employing a membrane model containing adequate concentrations of cholesterol showed that CSA was completely dehydrated near the center of the membrane and could not permeate through the center of the membrane unless a closed structure was adopted. These results are consistent with those previously published.

Based on these results, we predicted the PMFs and permeability of 18 ten-residue peptides using a POPC membrane model containing 40 and 50 mol % cholesterol. As a result, we obtained correlation coefficients of *R* > 0.8. The results demonstrate that our protocol is adequately capable of predicting the membrane permeation process of cyclic peptides and can identify peptides with high membrane permeability.

## Methods

### Target Peptides

**Figure 6:**
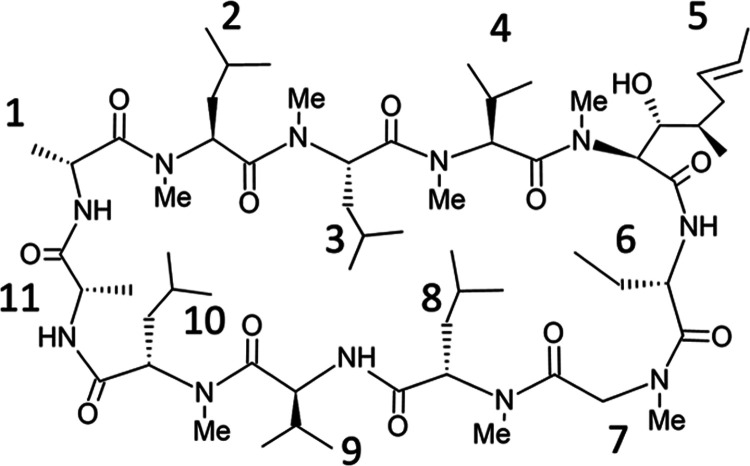
Chemical structure of cyclosporine A.

**Figure 7:**
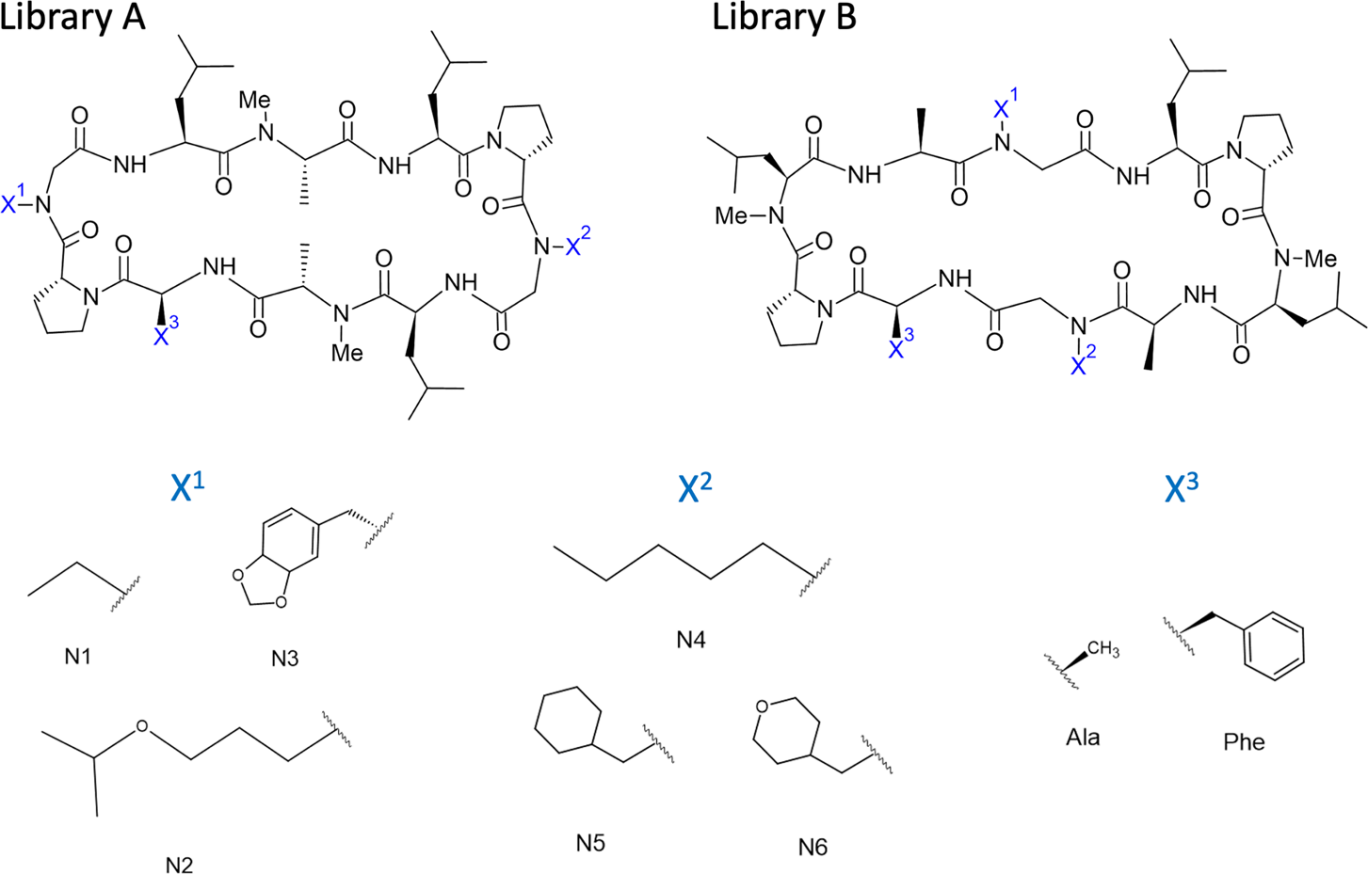
Chemical structure of peptides for Furukawa:MDCK and Furukawa:PAMPA data.

In this study, cyclosporine A and 10-residue peptides, whose experimental membrane permeability values have been provided by Furukawa et al.,^32^ we call them Furukawa data, were used in the calculations. The Furukawa data were chosen as the target of this study because it was a unique data set showing many experimental values of membrane permeability for peptides with more than or equal to 10 residues, which were almost nonexistent at the beginning of this project. The structure of cyclosporine A is shown in Figure 6, while the structure of the Furukawa data is shown in Figure 7. The net charge for all peptides is 0. For the Furukawa data, both MDCK assay data and PAMPA assay data are available and we call them Furukawa:MDCK and Furukawa:PAMPA data. Furukawa:MDCK data consist of six peptides listed in Table 3. Although Furukawa:PAMPA data consist of 18 peptides in both library A and library B, we target the peptides with AlogP < 4 in library B because of two reasons. First, the computational cost was too high to perform simulations for all of the peptides. Second, our previous study showed that some factors that cannot be reproduced by the simulation along the lipid bilayer have a dominant effect on the membrane permeability for highly hydrophobic peptides,^39^ and we noticed that the library A of the Furukawa:PAMPA data have similar characteristics to the hydrophobic peptides. The selected targets are shown in Tables 3 and 4.

**Table 3:** Substituents of Furukawa:MDCK Data^a^

library	index	X^1^	X^2^	X^3^
A	03	N1	Ala	N6
A	08	N1	Phe	N5
A	09	N1	Phe	N6
B	03	N1	Ala	N4
B	08	N1	Phe	N5
B	09	N1	Phe	N6

a B03, B08, and B09 are also included in Furukawa:PAMPA data.

**Table 4:** Substituents of Furukawa:PAMPA Data

library	index	X^1^	X^2^	X^3^
B	01	N1	Ala	N4
B	02	N1	Ala	N5
B	04	N2	Ala	N4
B	05	N1	Phe	N4
B	06	N2	Ala	N5
B	07	N2	Ala	N6
B	10	N3	Ala	N4
B	11	N3	Ala	N5
B	12	N3	Ala	N6
B	13	N2	Phe	N4
B	15	N2	Phe	N6
B	18	N3	Phe	N6

### Model Membrane

In this study, model membranes consisting of 1-palmitoyl-2-oleoyl-*sn*-glycero-3-phosphocholine (POPC) with different concentrations of cholesterol were used to predict the membrane permeation process of cyclic peptides and membrane permeability. Six membrane models with different cholesterol concentrations, 0–50 mol % at 10 mol % intervals, were prepared. Each membrane model was prepared using the Charmm-GUI server,^57^ without an additional equilibration process. The composition of each system is presented in Table 5.

**Table 5:** Composition of Model Membranes^a^

%Chol	#POPC	#Chol	#Wat	#Na^+^	#Cl^–^	area/lip
0	102	0	6240	13	13	63.3
10	108	12	7200	16	16	62.2
20	96	24	7200	16	16	55.8
30	84	36	7200	16	16	50.0
40	72	48	7200	16	16	46.0
50	60	60	7200	16	16	43.1

a %Chol corresponds to the mol. percentage of cholesterol determined based on the number of molecules. #POPC, #Chol, #Wat, #Na^+^, and #Cl^–^ correspond to the number of POPC molecules, cholesterol molecules, water molecules, Na^+^ ions, and Cl^–^ ions, respectively. Area/Lip corresponds to the area per lipid (Å^2^) estimated from the trajectories of the steered MD.

### Protocol for Simulating the Membrane Permeation Process and Estimating Membrane Permeability

In this study, MD simulations using the REST/REUS method^50^ were applied to predict the membrane permeability of cyclic peptides. The REST method accelerates the sampling of solute molecules by simultaneously running multiple simulations in which only the solute molecules were set at different temperatures to the solvent, usually called a replica, and periodically exchanging the temperature of the solute molecule between replicas.^58^ The REUS method is a technique for sampling throughout the reaction coordinate by simultaneously running multiple umbrella sampling (US) simulations that are positionally constrained to the arbitrary position to be sampled and periodically exchanging the constrained positions between them.^50^ The REST/REUS method is a technique used to effectively sample the conformation of solute molecules along the entire reaction coordinate by simultaneously performing the REST and REUS methods. The membrane permeability of the peptide was estimated based on a slightly modified inhomogeneous solubility–diffusion model (ISDM), subsequently mentioned in the Methods section.^15,39^ Our protocol assumes that the PMF on both sides across the center of the membrane is symmetric, and the PMF calculated on one side of the membrane is used to supplement the overall PMF. All MD simulations were performed using the GPU-accelerated PMEMD module (pmemd.cuda) of the AMBER 20 software package.^59^ The peptides were parameterized using Amber10: Extended Huckel Theory (EHT) parameter set in Molecular Operating Environment (MOE) from Chemical Computing Group.^60^ The ff10 bonded parameters were used for all atoms, which are recognized as Amber10 types.^61^ The EHT parameters were used for atoms not recognized as Amber10 types. The EHT method uses a 2D quantum mechanical approach based on the MAB method^62^ to derive the stretch, bend, and torsion parameters for any atom. The Amber10 VDW parameters were used for all atoms. Amber10 partial charges were used for atoms recognized as Amber10 types. MMFF94 partial charges^63^ were used for atoms not recognized as Amber10 types. POPC molecules were parameterized using Lipid 17 force fields.^59,64^ We used the TIP3P model as the water molecule.^65^ The detailed protocol is described below:

(1) To prepare the initial coordinates of the REST/REUS method, steered MD^66^ combined with the solute tempering (ST) method^58^ was performed. That is, the potential energy was used for this process, which was obtained by scaling the nonbonding interaction energy and dihedral energy of the peptide to 0.143. With this parameter, the temperature of the peptide corresponds to 2100 K when the temperature of the system was set to 300 K. The application of Steered MD based on the ST method was a strategy to obtain as many diverse peptide conformations as possible to be used as the initial structure for the REST/REUS method. Methylated peptide bonds can be easily rotated at 2100 K, as shown in Figure S1a. First, an arbitrary structure of the peptide was placed in bulk water of the simulation box so that the center of mass of the nitrogen atoms of peptide bonds was located at *z* = 40.0 Å. Composition of the molecules in each simulation box is described in the Model Membrane section. The initial structure was minimized for 10,000 steps, where the first 5000 steps used the steepest descent method and the remaining 5000 steps used the conjugate gradient method. In this process, the peptide and lipid molecules were restrained with harmonic potential, at a 2 kcal/mol/Å^2^ force constant. The systems were then heated from 0 to 100 K within 20 ps using constant-volume Langevin dynamics. In this process, the peptide and lipid molecules were restrained with harmonic potential, the force constant of which was 1 kcal/mol/Å^2^. Thereafter, the temperature was increased to 300 K within 100 ps in the isothermal–isobaric (NPT) ensemble with semi-isotropic pressure scaling. The pressure was controlled using a Berendsen barostat and was maintained at 1 bar. The force constant of the harmonic potential for the restrained peptide and lipid molecules decreased to 0.1 kcal/mol/Å^2^. The systems were equilibrated for 10 ns with positional restraint at *z* = 40.0 Å. Next, peptides were pulled based on steered MD^66^ from *z* = 40.0 Å, a position slightly beyond the reaction coordinate for the REST/REUS simulation, to −5.0 Å, a position slightly beyond the center of the membrane. A pulling rate of 0.56 Å/ns and a force constant of 3.0 kcal/mol/Å^2^ were used for CSA, and a pulling rate of 0.25 Å/ns and a force constant of 3.0 kcal/mol/Å^2^ were used for Furukawa data.
(2) We explored the phase space using the REST/REUS method.^50^ The REST/REUS simulation was carried out with 28 windows with different restraint centers of a harmonic potential and eight windows with different temperatures of the solute, that is, 28 × 8 replicas. For the section from *z* = 0 to 6 Å, near the center of the bilayer, the restraint center and force constant were set to 1.0 Å interval and 1.5 kcal/mol/Å^2^, respectively. For the section from *z* = 6.0 to 37.5 Å, the restraint center and force constant were set to 1.5 Å interval and 0.5 kcal/mol/Å^2^, respectively. The initial coordinates of eight temperature replicas for each restraint parameter were randomly selected from the steered MD trajectory, where the peptide was located at ±0.4 Å from the restraint center for the REST/REUS simulation. The simulation consisted of a 200 ns equilibration followed by a 300 ns production run. However, for the calculation of cyclosporine A using the membrane model with cholesterol concentrations of 10, 20, and 40 mol %, where the degree of convergence of PMF was poor, and the equilibration process was extended to 300 ns. Exchanges between adjacent replicas were attempted every 10 ps using the Metropolis scheme. The temperatures of the peptide for each of the eight temperature replica were set to 300, 340, 390, 455, 540, 645, 785, and 980 K to maintain an exchange rate of approximately 10–20%. The maximum temperature of 980 K was sufficient for cis–trans isomerization of the ω angle of the peptide bonds on N-substituted peptides and proline. After the REST/REUS simulation, the PMF was estimated based on WHAM using the trajectory of the lowest temperature replicas.^67−70^ The error bars were determined as the standard deviation of PMFs calculated independently of the trajectories of the first 150 ns and second 150 ns.
(3) We estimated the local diffusion constants based on 20 ns US simulations^71^ with the same coordinate intervals as the REUS simulation, but with the force constant of all restraint potentials set to 2.5 kcal/mol/Å^2^. The local diffusion constant, *D*(*z*), was calculated (eq 1).
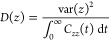
1where *C*_*zz*_(*t*) = ⟨δ_*z*_(0)δ_*z*_(*t*)⟩ is the autocorrelation function of the *z-*position of the peptide in the PMF window.^72^ The US is performed two times using the coordinates at 150 and 300 ns of the lowest temperature replica of the REST/REUS simulation.
(4) We estimated membrane permeability based on the modified ISDM.^15^ Local resistance values *R*(*z*) was calculated using eq 2.
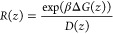
2where β = 1/*k*_B_*T* is the inverse temperature, *k*_B_ is the Boltzmann constant, and *T* is the thermodynamic temperature. Δ*G*(*z*) represents the difference between *G*(*z*) and the minimum point of PMF, not *G*(*z*) and the bulk (*z* = 37.5 Å) value. The integration of the *R*(*z*) profile allows for the calculation of the overall permeation coefficient (eq 3).
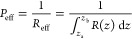
3Based on a previous study,^39^ two types of *P*_eff_ values were defined: *P*_flip_ and *P*_out_. To estimate *P*_flip_, *z*_a_ and *z*_b_ were defined as the minimum point on PMF and *z* = 0 Å, respectively. Furthermore, to estimate *P*_out_, *z*_a_ and *z*_b_ were defined as the minimum point on the PMF, and *z* = 37.5 Å, respectively. When the minimum of the PMF is located at a position greater than *z* = 30 Å, *P*_out_ cannot be defined and was ignored. Although this model was applied to predict the membrane permeability for more than 100 six- and eight-residue peptides and predicted reasonable values,^39^ there are two limitations. First, the model cannot reproduce the phenomenon sometimes observed on hydrophobic peptides that the logarithm of the membrane permeability is inversely proportional to their log *P* values. This may be due to the membrane permeability of hydrophobic peptides being determined by factors other than the process of permeation through the lipid bilayer, such as the diffusion rate in UWL and low solubility.^25,26,28,29^ Second, the method used in this study relies on PMF and diffusion coefficients along one-dimensional reaction coordinates. Therefore, it is incapable of predicting plausible membrane permeation coefficients when motion in a direction orthogonal to the reaction coordinate is associated with the rate-limiting process of membrane permeation. Such situations may arise when the rate-limiting process of membrane permeation depends on changes in the orientation of the peptide within the membrane, or on changes in the conformation of the peptide.
  For each peptide simulation, 224 GPU boards were simultaneously employed for performing REST/REUS simulation, with 28 × 8 replicas. A 500 ns run, consisting of 200 ns equilibration followed by a 300 ns production run, was completed in approximately 90 h using NVIDIA P100 GPUs. Thus, the total calculation in this study took approximately 860 thousand GPU h.

### Analysis

#### Polar Surface Area

The polar surface area is estimated as a solvent-accessible surface area around N and O atoms in the main and side chains with the LCPO algorithm^73^ by means of the cpptraj module of AmberTools20.^59^

#### Dihedral Angle Principal Component Analysis

In the dihedral angle principal component analysis, the sine and cosine values of the dihedral angles (ψ,ϕ,ω) for all snapshots of the peptide backbone were used for determining the eigenvectors^52^ by means of the cpptraj module of AmberTools20.^59^

## Data and Software Availability

Parameterization and initial conformational sampling of peptides were performed using MOE (2019.0104) (https://www.chemcomp.com/index.htm).^60^ All molecular dynamics simulations were performed using AMBER 20 software package (https://ambermd.org/).^59^ PMF was estimated using WHAM:version 2.0.9 (http://membrane.urmc.rochester.edu/?page_id=126^70^ The structures of CSA and Furukawa data are shared as a zip file at the supporting information.

## Supporting Information Available

mol2 files of CSA and Furukawa data (ZIP)
Profiles of the ω angle of CSA in the trajectory of steered MD based on the solute tempering (ST) method; free energy profiles along the reaction coordinate *z*; AlogP and experimentally assessed and calculated membrane permeability values; angle between the principal axis of inertia and the reaction coordinate *z*; representative snapshots of the CSA; area per lipid values for the data of CSA at 10 mol % cholesterol concentration; diffusion coefficient along the reaction coordinate *z*; free energy profiles along the first and second principal components obtained from dPCA; content of closed structures of CSA depending on the cholesterol concentration of the membrane; scatter plot illustrating the experimental values of membrane permeability for the Furukawa:MDCK data and calculated values obtained using a cholesterol-free POPC membrane; average number of hydrogen bonds between peptide and water molecules against reaction coordinate *z* for six-residue peptides (PDF)
