# Supplementary material for: Lipid Composition Is Critical for Accurate Membrane Permeability Prediction of Cyclic Peptides by Molecular Dynamics Simulations
Source: J Chem Inf Model. 2022 Sep 2;62(18):4549–60. doi: 10.1021/acs.jcim.2c00931 (PMC9516681; doi:10.1021/acs.jcim.2c00931)
Supplement: Supplementary file 1 — ci2c00931_si_001.pdf [file ci2c00931_si_001.pdf]

SUPPORTING INFORMATION

Lipid composition is critical for accurate membrane permeability prediction of cyclic peptides by molecular dynamics simulations

Masatake Sugita[1,2], Takuya Fujie[1,2], Keisuke Yanagisawa[1,2], Masahito Ohue[1,2], Yutaka Akiyama[1,2,]*

[1]Department of Computer Science, School of Computing, Tokyo Institute of Technology, W8-76, 2-12-1 Ookayama, Meguro-ku, Tokyo 152-8550, Japan
[2]Middle-Molecule IT-based Drug Discovery Laboratory (MIDL), Tokyo Institute of Technology, W8-76, 2-12-1 Ookayama, Meguro-ku, Tokyo 152-8550, Japan

*Corresponding author

Yutaka Akiyama
Email: akiyama@c.titech.ac.jp

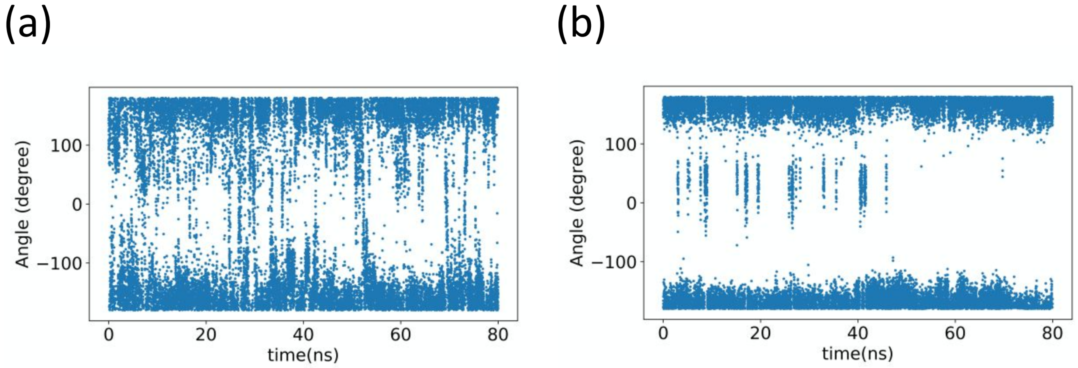

Figure S1. Profiles of ω angle between residue 1 and 2 in the trajectory of steered MD based on the solute tempering (ST) method. (a) Data from simulation trajectory performed at 2,100 K to obtain the initial structure for the REST/REUS method, and (b) data from simulation trajectory performed at 980 K, corresponding to the highest temperature replica of the REST/REUS method A steered MD at 980 K was performed to confirm that methylated peptide bonds can be rotated at this temperature.

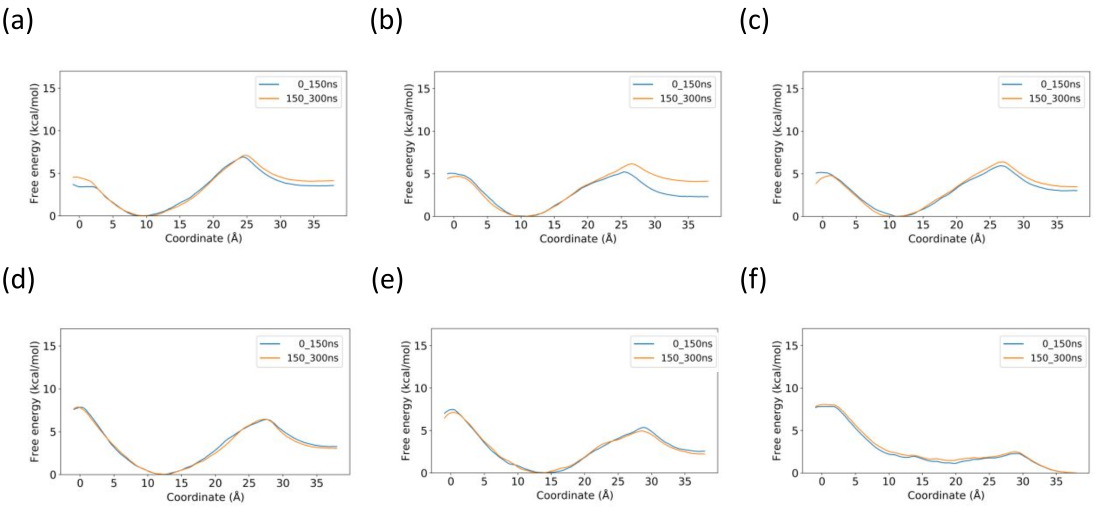

Figure S2. Free energy profiles estimated every 150ns for the simulation using the model membrane with (a) 0%, (b) 10%, (c) 20%, (d) 30%, (e) 40%, and (f) 50% mole fractions of cholesterol

Table S1. AlogP and experimentally assessed and calculated membrane permeability values of cyclosporine A (CSA) against membrane models with different cholesterol concentration

Cholesterol contents	AlogP	$\log P_{PAMPA}$	$\log P_{MDCK}$	$\log P_{ISDM\_mod}$	$\log P_{flip}$	$\log P_{out}$	MAE_{PMF}
0%	4.33	-5.30	-5.85 ± 0.06	-3.98 ± 0.07	-1.86 ± 0.42	-3.98 ± 0.07	0.32
10%	4.33	-5.30	-5.85 ± 0.06	-3.01 ± 0.98	-2.55 ± 0.13	-3.01 ± 0.98	0.68
20%	4.33	-5.30	-5.85 ± 0.06	-3.45 ± 0.17	-2.66 ± 0.15	-3.45 ± 0.17	0.32
30%	4.33	-5.30	-5.85 ± 0.06	-4.37 ± 0.05	-4.37 ± 0.05	-3.69 ± 0.01	0.17
40%	4.33	-5.30	-5.85 ± 0.06	-4.17 ± 0.08	-4.17 ± 0.08	-2.74 ± 0.14	0.20
50%	4.33	-5.30	-5.85 ± 0.06	-5.23 ± 0.39	-5.23 ± 0.39	-	0.21

A single value for AlogP is listed because the value depends only on the chemical structure of the peptide and is independent of cholesterol concentration. Since there are no experiments reporting membrane permeability changes as a function of cholesterol concentration for CSA, a single experimental value for $\log P_{PAMPA}$ and $\log P_{MDCK}$ are also listed. The hyphen indicates that P_{out} cannot be defined because the minimum of the potential of mean force (PMF) is located at a position greater than z = 30 Å. $\log P_{PAMPA}$ corresponds to the average value of permeability reported in three studies. [1-3] Error bars for $\log P_{PAMPA}$ were not included, as these values were not provided in some of the referenced experimental data. The experimental data of $\log P_{MDCK}$ was obtained from the literature of Naylor et al. [4] MAE_{PMF} denotes the mean absolute error (MAE), $\sum_{i=1}^{n} |F_a(z_i) - F_b(z_i)|/n$, between two PMFs obtained from the trajectories of 0–150 ns and 150–300 ns, respectively, of the production run.

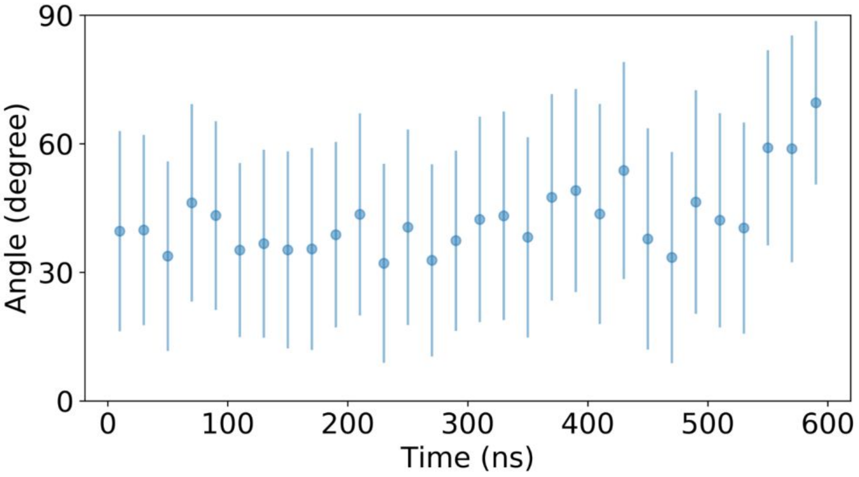

Figure S3. The angle between the principal axis of inertia corresponding to the maximum eigenvalue and the reaction coordinate z at z = 27 Å

The plot and error bars show the mean value every 25 ns and the standard deviation over that interval.

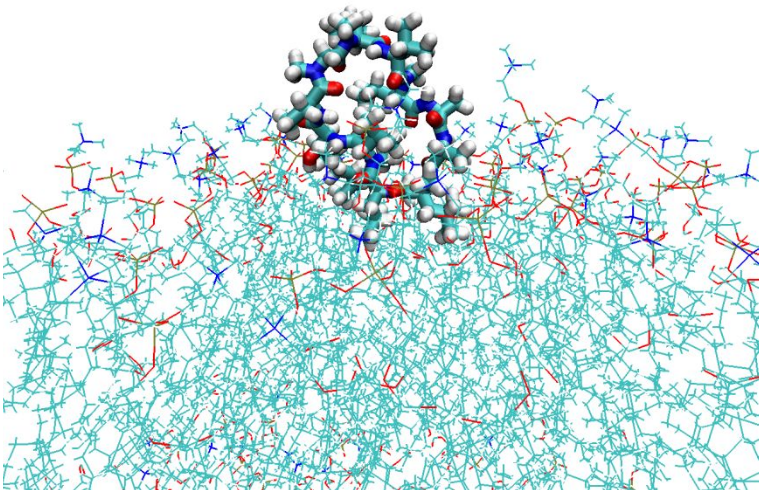

Figure S4. A representative snapshot of the CSA anchored to the membrane at z = 27 Å

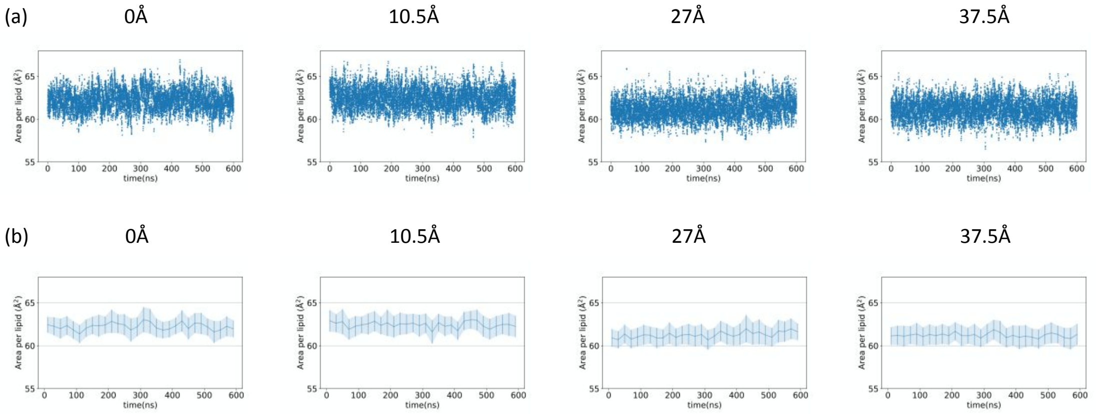

Figure S5. (a) Area per lipid values for the data at 10 mol% cholesterol concentration plotted against time and (b)the mean value every 25 ns and the standard deviation over that interval

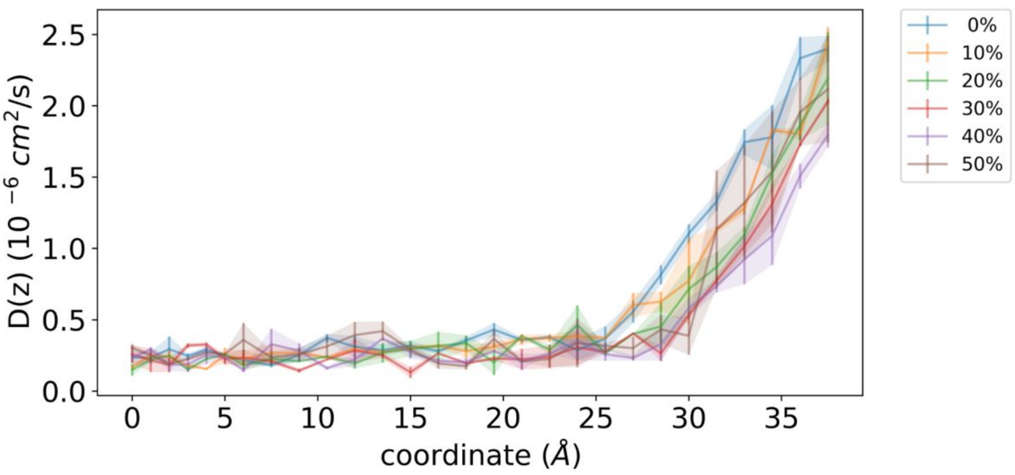

Figure S6. Diffusion coefficient along the reaction coordinate *z* estimated by using the model membrane with 0 to 50 % mole fractions of cholesterol

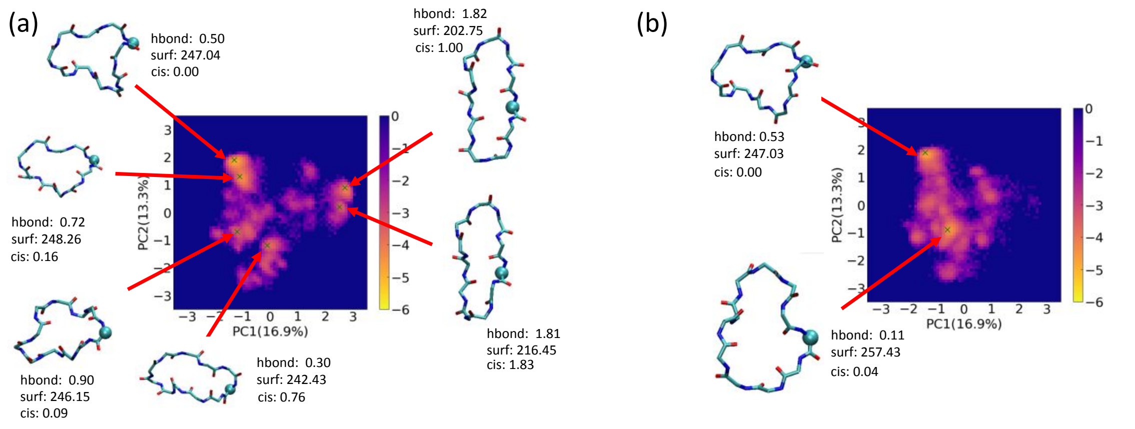

Figure S7. Representative snapshots on the free energy minima for the simulation with cholesterol-free membrane at (a) *z* = 0–5 Å and (b) *z* = 30–37.5 Å

Average polar surface area, hydrogen bond number, and number peptide bonds with cis-type ω dihedral angle are shown next to the snapshots. The average values for each type were calculated using all snapshots within ±3 along the eigen vectors from the free energy minima marked with crosses.

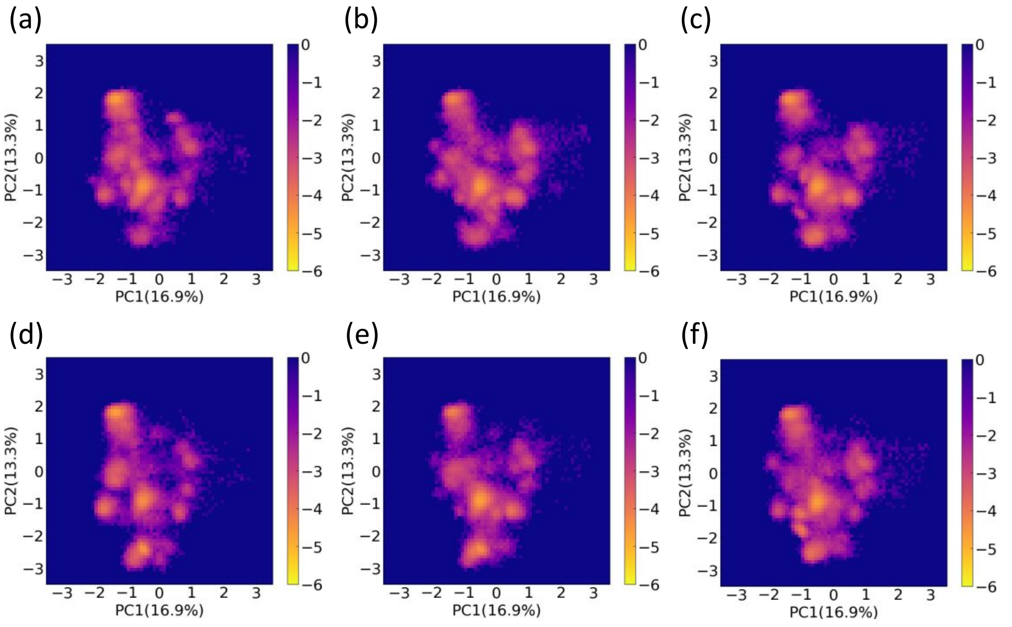

Figure S8. Free energy profiles that are calculated based on projected trajectories at *z* = 30–37.5 Å with model membranes containing (a) 0%, (b) 10%, (c) 20%, (d) 30%, (e) 40%, and (f) 50% cholesterol on the eigen vectors of the first and second principal components obtained from dPCA

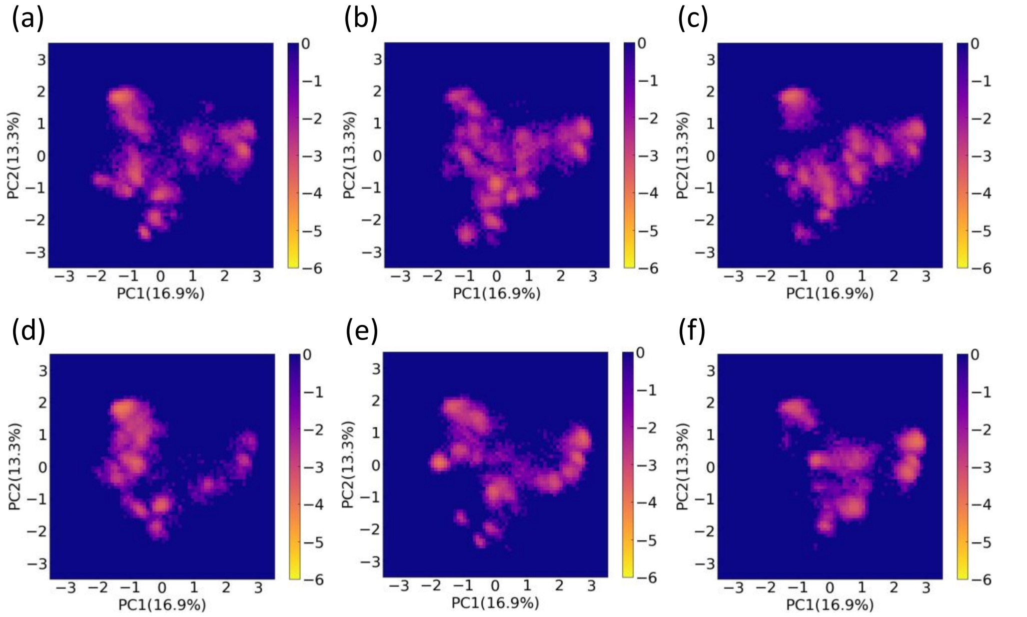

Figure S9. Free energy profiles that are calculated based on projected trajectories at z = 9–10.5 Å with model membranes containing (a) 0%, (b) 10%, (c) 20%, (d) 30%, (e) 40%, and (f) 50% cholesterol on the eigen vectors of the first and second principal components obtained from dPCA

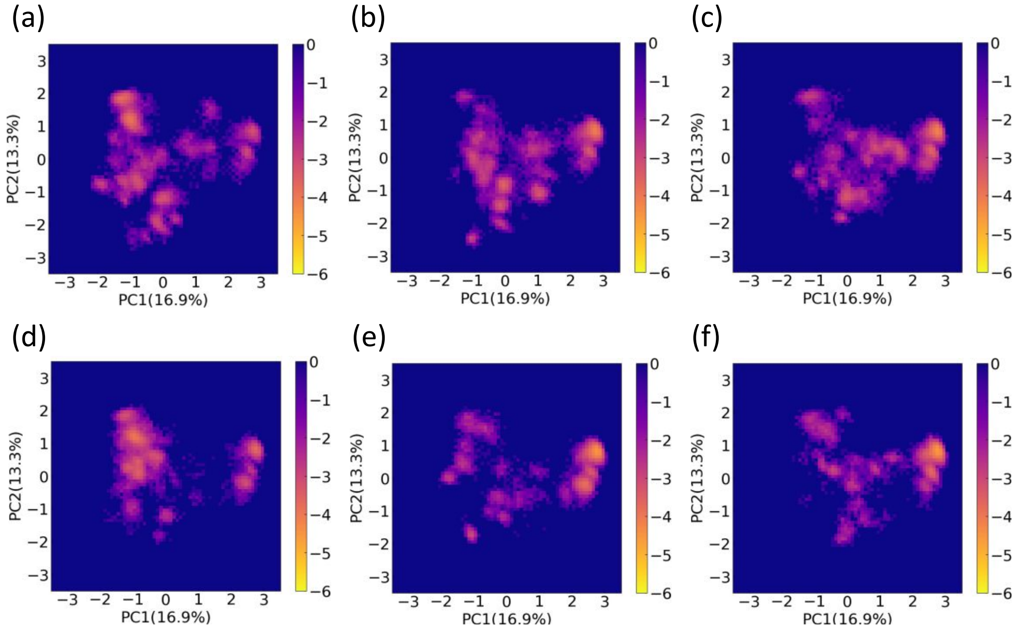

Figure S10. Free energy profiles that are calculated based on projected trajectories at z = 4–5 Å with model membranes containing (a) 0%, (b) 10%, (c) 20%, (d) 30%, (e) 40%, and (f) 50% cholesterol on the eigen vectors of the first and second principal components obtained from dPCA

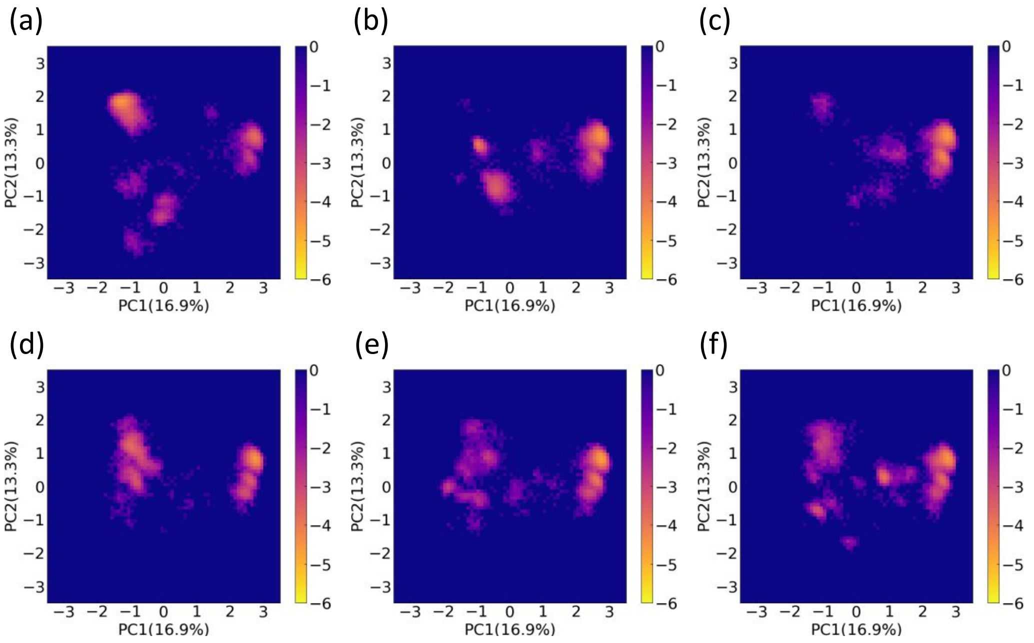

Figure S11. Free energy profiles that are calculated based on projected trajectories at z = 0–1 Å with model membranes containing (a) 0%, (b) 10%, (c) 20%, (d) 30%, (e) 40%, and (f) 50% cholesterol on the eigen vectors of the first and second principal components obtained from dPCA

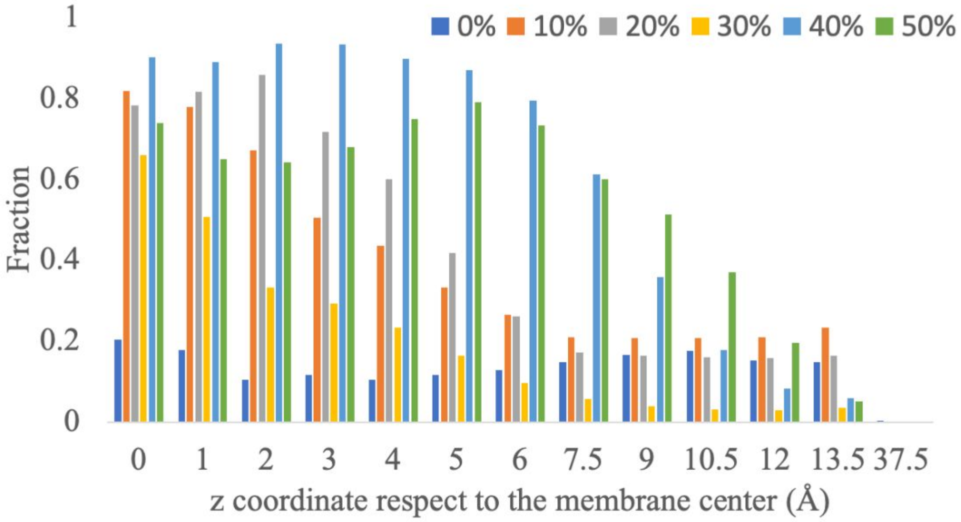

Figure S12. Content of closed structures depending on the cholesterol concentration of the membrane. The bars were color-coded by cholesterol concentration

The legend shows the colors associated with the percentages (mole fraction) of the cholesterol.

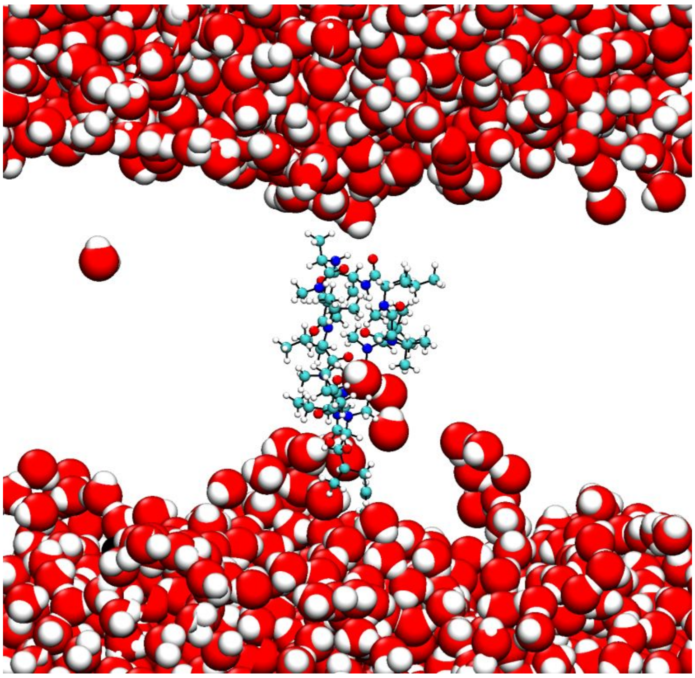

Figure S13. A snapshot obtained from a simulation using a cholesterol-free membrane model. The water molecules are shown with space fill model, and the cyclosporine A is shown in ball-and-stick model

The membrane model is not shown.

Table S2. AlogP and experimentally assessed and calculated membrane permeabilities of Furukawa:MDCK and Furukawa:PAMPA data using a membrane model containing 40% cholesterol

Peptide Name	AlogP	$\log P_{PAMPA}$	$\log P_{MDCK}$	$\log P_{ISDM\_mod}$	$\log P_{flip}$	$\log P_{out}$	MAE_{PMF}
A03	0.394	-5.63 ± 0.10	-6.4 ± 0.11	-7.59 ± 0.30	-7.59 ± 0.30	-	0.18
A08	3.719	-6.34 ± 0.04	-4.61 ± 0.06	-4.41 ± 0.05	-4.41 ± 0.05	-3.05 ± 0.17	0.29
A09	1.95	-5.35 ± 0.06	-5.51 ± 0.07	-5.50 ± 0.20	-5.50 ± 0.20	-	0.56
B01	1.76	-6.2 ± 0.03	-	-3.36 ± 0.04	-3.36 ± 0.04	-	0.56
B02	2.16	-5.82 ± 0.03	-	-3.81 ± 0.17	-3.81 ± 0.17	-1.24 ± 0.39	0.39
B03	0.394	-7.76 ± 0.00	-6.52 ± 0.00	-7.22 ± 1.19	-7.22 ± 1.19	-	0.75
B04	2.069	-5.75 ± 0.03	-	-4.57 ± 0.37	-4.57 ± 0.37	-	0.40
B05	3.316	-5.3 ± 0.04	-	-2.94 ± 0.05	-2.94 ± 0.05	-1.58 ± 0.34	0.38
B06	2.473	-5.78 ± 0.04	-	-4.03 ± 0.09	-4.03 ± 0.09	-	0.37
B07	0.703	-7.44 ± 0.00	-	-8.02 ± 0.23	-8.02 ± 0.23	-	0.40
B08	3.719	-5.27 ± 0.04	-4.82 ± 0.15	-3.21 ± 0.12	-3.21 ± 0.12	-2.06 ± 0.15	0.24
B09	1.95	-6.85 ± 0.03	-6.4 ± 0.11	-6.63 ± 0.23	-6.63 ± 0.23	-	0.28
B10	2.762	-5.69 ± 0.03	-	-4.90 ± 0.19	-4.90 ± 0.19	-1.85 ± 0.07	0.24
B11	3.166	-5.53 ± 0.04	-	-4.31 ± 0.16	-4.31 ± 0.16	-1.59 ± 0.39	0.40
B12	1.397	-6.86 ± 0.03	-	-7.51 ± 0.35	-7.51 ± 0.35		0.62
B13	3.625	-5.30 ± 0.05	-	-4.28 ± 0.32	-4.28 ± 0.32	-1.59 ± 0.15	0.27
B15	2.259	-6.48 ± 0.03	-	-6.73 ± 0.12	-6.73 ± 0.12	-	0.16
B18	2.953	-6.27 ± 0.03	-	-7.18 ± 0.08	-7.18 ± 0.08	-	0.20

The hyphen indicates that P_{out} cannot be defined because the minimum of the PMF is located at a position greater than z = 30 Å. $\mathrm{MAE_{PMF}}$ denotes the mean absolute error (MAE), $\sum_{i=1}^{n}|F_a(z_i) - F_b(z_i)|/n$, between two PMFs obtained from the trajectories of 0–150 ns and 150–300 ns, respectively, of the production run.

Table S3. AlogP and experimentally assessed and calculated membrane permeabilities of Furukawa:MDCK and Furukawa:PAMPA data using a membrane model containing 50% cholesterol

Peptide Name	AlogP	$\log P_{PAMPA}$	$\log P_{MDCK}$	$\log P_{ISDM\_mod}$	$\log P_{flip}$	$\log P_{out}$	MAE_{PMF}
A03	0.394	-5.63 ± 0.10	-6.4 ± 0.11	-8.87 ± 0.04	-8.87 ± 0.04	-	0.25
A08	3.719	-6.34 ± 0.04	-4.61 ± 0.06	-3.26 ± 0.03	-3.26 ± 0.03	-1.12 ± 0.16	0.35
A09	1.95	-5.35 ± 0.06	-5.51 ± 0.07	-6.74 ± 0.06	-6.74 ± 0.06	-	0.23
B01	1.76	-6.2 ± 0.03	-	-4.61 ± 0.56	-4.61 ± 0.56	-	0.29
B02	2.16	-5.82 ± 0.03	-	-5.31 ± 0.18	-5.31 ± 0.18	-	0.33
B03	0.394	-7.76 ± 0.00	-6.52 ± 0.00	-8.87 ± 0.06	-8.87 ± 0.06	-	0.11
B04	2.069	-5.75 ± 0.03	-	-5.39 ± 0.55	-5.39 ± 0.55	-	0.60
B05	3.316	-5.3 ± 0.04	-	-4.62 ± 0.67	-4.62 ± 0.67	-	0.76
B06	2.473	-5.78 ± 0.04	-	-5.85 ± 1.03	-5.85 ± 1.03	-	0.83
B07	0.703	-7.44 ± 0.00	-	-9.88 ± 0.19	-9.88 ± 0.19	-	0.21
B08	3.719	-5.27 ± 0.04	-4.82 ± 0.15	-3.86 ± 0.05	-3.86 ± 0.05	-	0.21
B09	1.95	-6.85 ± 0.03	-6.4 ± 0.11	-7.54 ± 0.46	-7.54 ± 0.46	-	0.48
B10	2.762	-5.69 ± 0.03	-	-5.68 ± 0.44	-5.68 ± 0.44	-	0.42
B11	3.166	-5.53 ± 0.04	-	-5.13 ± 0.07	-5.13 ± 0.07	-	0.27
B12	1.397	-6.86 ± 0.03	-	-9.97 ± 0.1	-9.97 ± 0.1	-	0.38
B13	3.625	-5.30 ± 0.05	-	-5.43 ± 0.23	-5.43 ± 0.23	-	0.24
B15	2.259	-6.48 ± 0.03	-	-8.81 ± 0.1	-8.81 ± 0.1	-	0.84
B18	2.953	-6.27 ± 0.03	-	-7.67 ± 0.13	-7.67 ± 0.13	-	0.28

The hyphen indicates that P_{out} cannot be defined because the minimum of the PMF is located at a position greater than z = 30 Å. MAE_{PMF} denotes the mean absolute error (MAE), $\sum_{i=1}^{n}|F_a(z_i) - F_b(z_i)|/n$, between two PMFs obtained from the trajectories of 0–150 ns and 150–300 ns, respectively, of the production run.

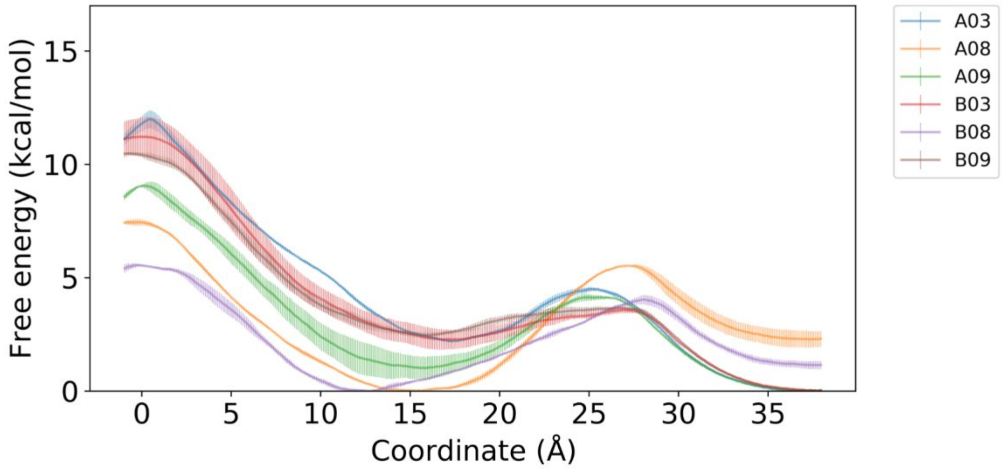

Figure 14. PMFs for the 10-residue peptides in the Furukawa:MDCK data by using the model membrane containing 40 mol% cholesterol

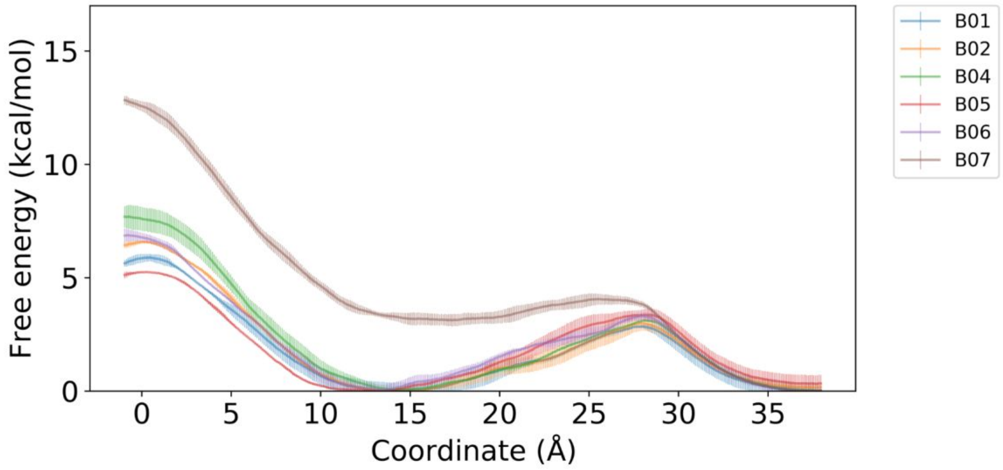

Figure 15. PMFs for the part of the Furukawa:PAMPA data using the model membrane containing 40 mol% cholesterol

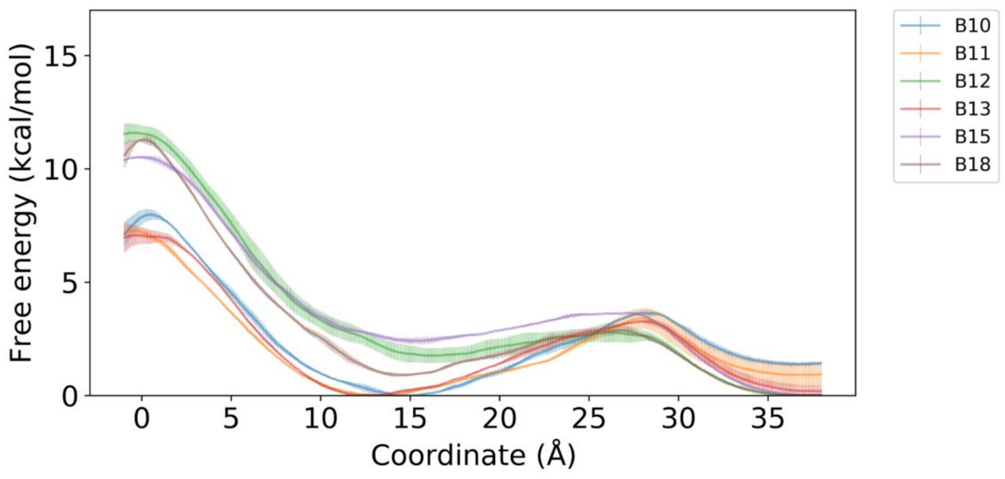

Figure 16. A continuation of PMFs for the part of the Furukawa:PAMPA data using the model membrane containing 40 mol% cholesterol

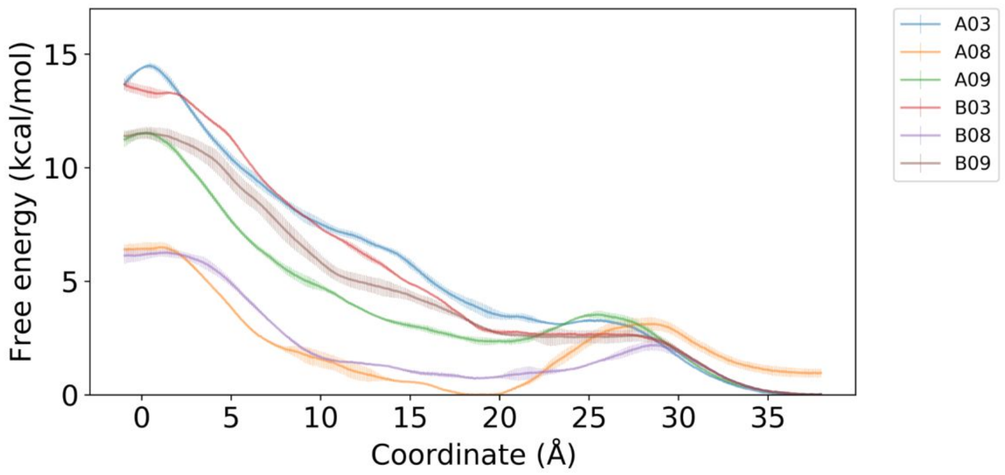

Figure S17. PMFs for the 10-residue peptides in the Furukawa:MDCK data by using the model membrane containing 50 mol% cholesterol

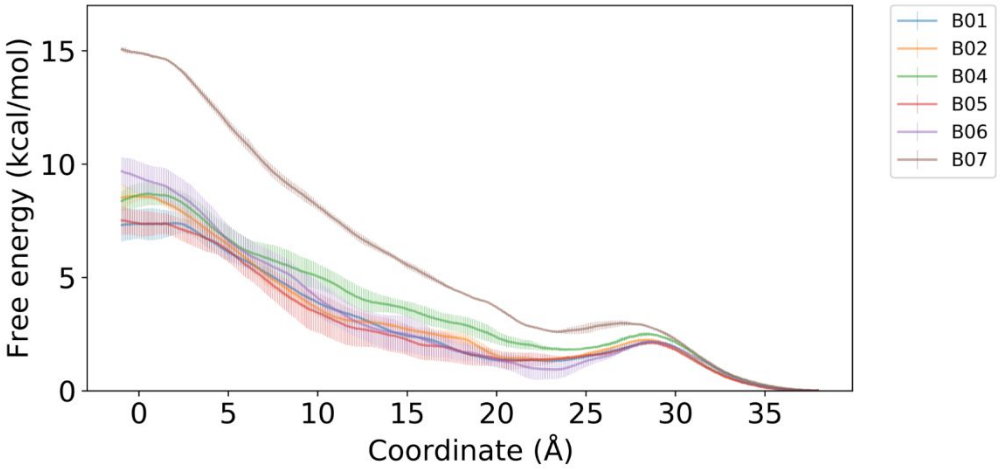

Figure S18. PMFs for the part of the Furukawa:PAMPA data by using the model membrane containing 50 mol% cholesterol

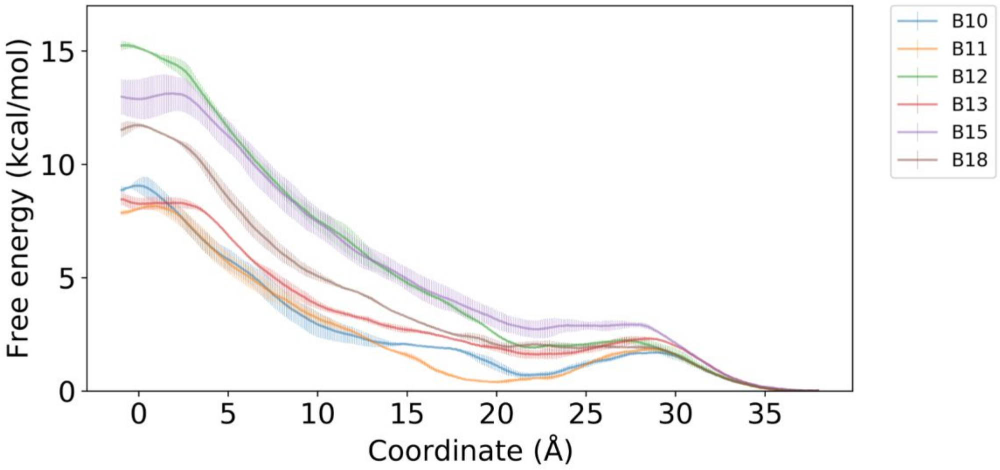

Figure S19. A continuation of PMFs for the part of the Furukawa:PAMPA data by using the model membrane containing 50 mol% cholesterol

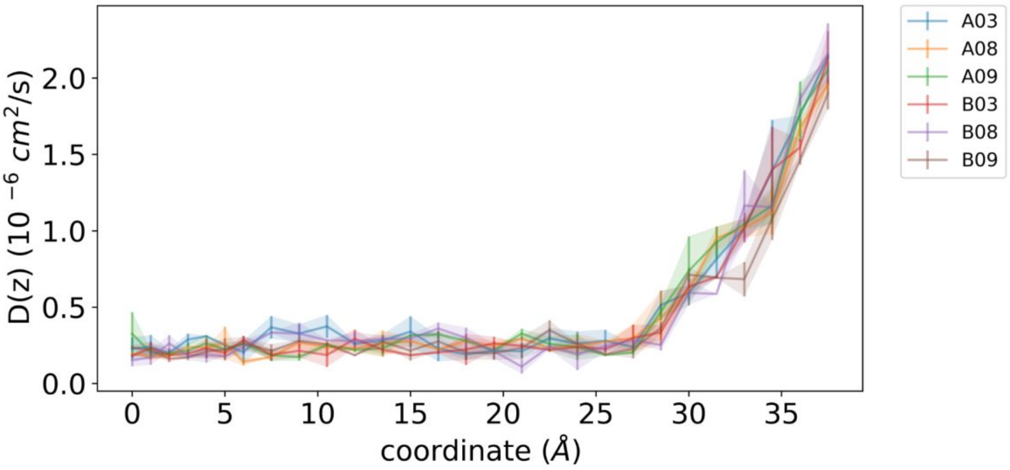

Figure S20. Diffusion coefficient along the reaction coordinate *z* for the 10-residue peptides in the Furukawa:MDCK data estimated by using the model membrane with 40 % mole fractions of cholesterol

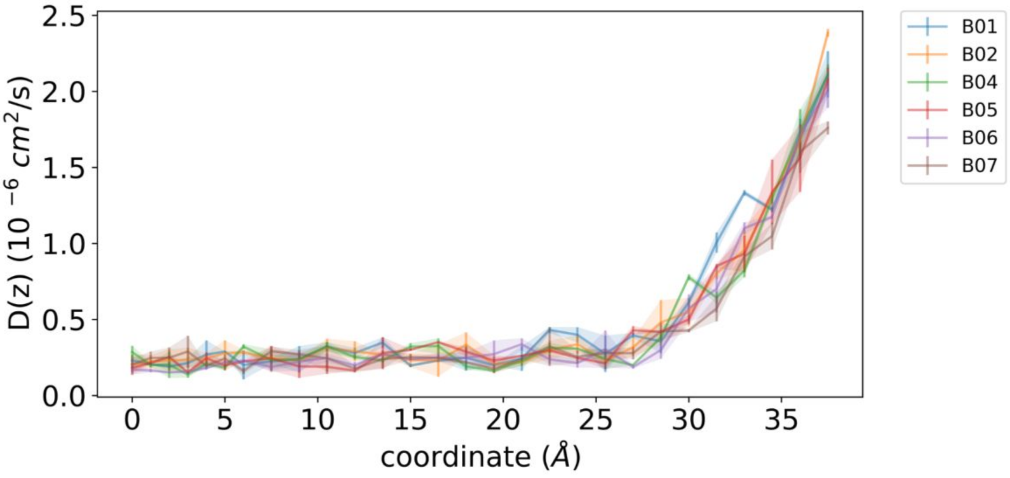

Figure S21. Diffusion coefficient along the reaction coordinate *z* for a part of the Furukawa:PAMPA data estimated by using the model membrane with 40% mole fractions of cholesterol

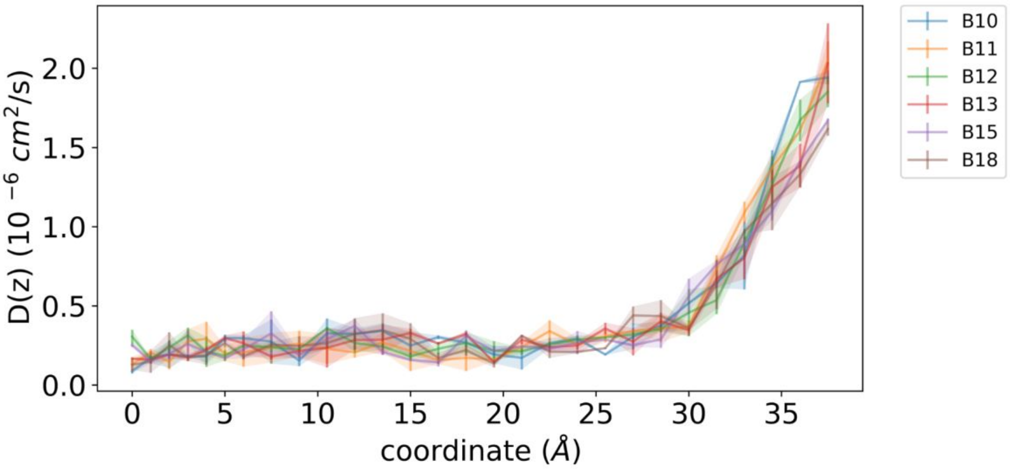

Figure S22. A continuation of diffusion coefficient along the reaction coordinate *z* for a part of the Furukawa:PAMPA data estimated by using the model membrane with 40% mole fractions of cholesterol

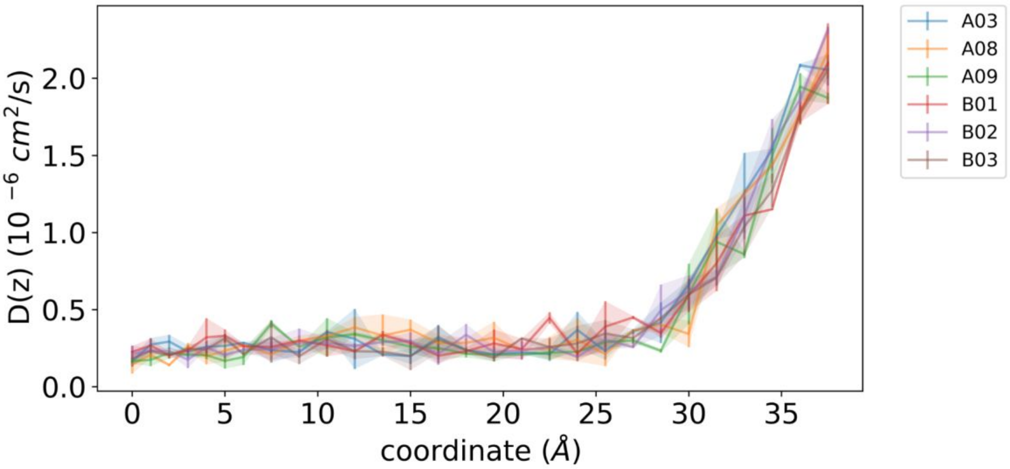

Figure S23. Diffusion coefficient along the reaction coordinate *z* for the 10-residue peptides in the Furukawa:MDCK data estimated by using the model membrane with 50% mole fractions of cholesterol

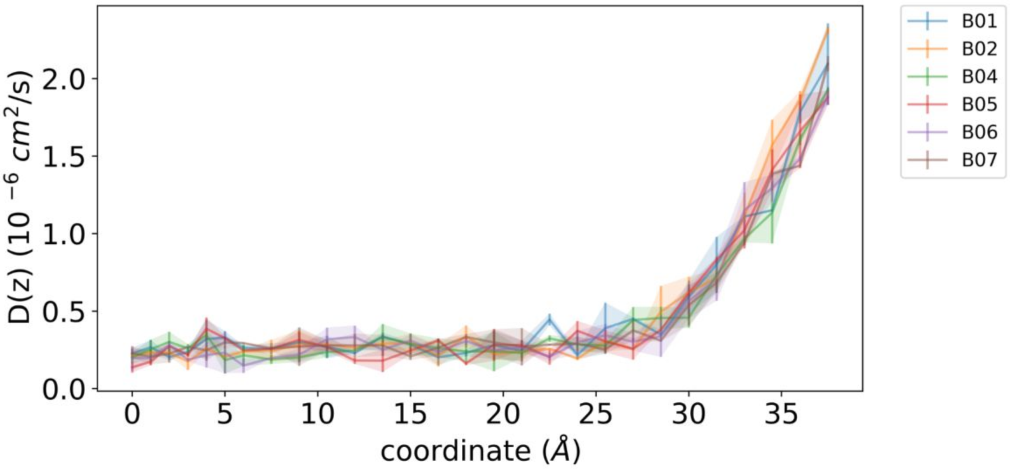

Figure S24. Diffusion coefficient along the reaction coordinate *z* for a part of the Furukawa:PAMPA data estimated by using the model membrane with 50% mole fractions of cholesterol

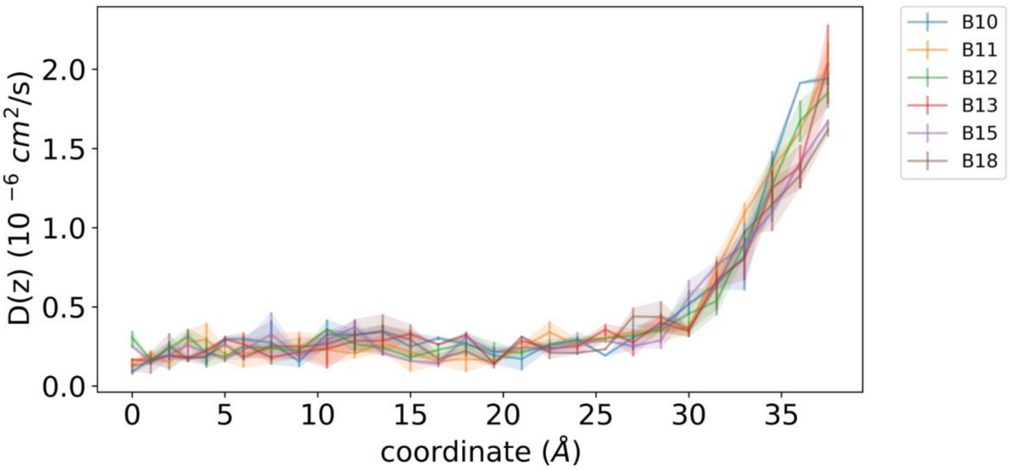

Figure S25. A continuation of diffusion coefficient along the reaction coordinate *z* for a part of the Furukawa:PAMPA data estimated by using the model membrane with 50% mole fractions of cholesterol

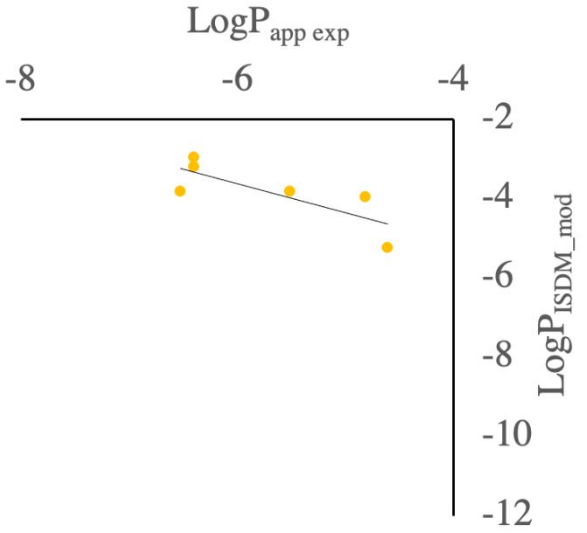

Figure S26. Scatter plot illustrating the experimental values of membrane permeability for the Furukawa:MDCK data and calculated values obtained using a cholesterol-free POPC membrane

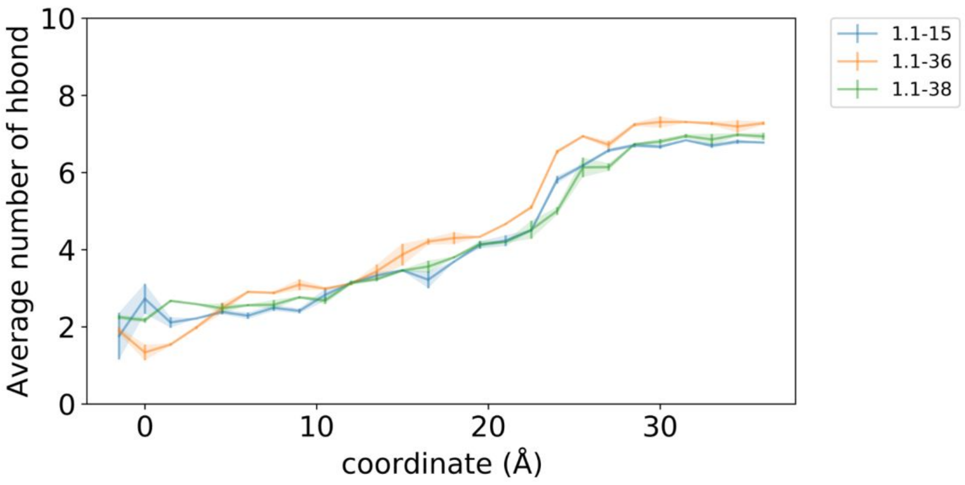

Figure S27. Average number of hydrogen bonds between peptide and water molecules against reaction coordinate *z*

The profile was obtained by analyzing the trajectory data from our previous study. The error bars are determined as the standard deviation of profiles calculated independently from the trajectories of the first 100 ns and second 100 ns. The three target peptides 1.1-15, 1.1-36, and 1.1-38 have AlogP values of 3.67, 3.47, and 4.34, respectively.[5]

REFERENCES

(1) Ahlbach, C. L.; Lexa, K. W.; Bockus, A. T.; Chen, V.; Crews, P.; Jacobson, M. P.; Lokey, R. S. Beyond Cyclosporine a: Conformation-Dependent Passive Membrane Permeabilities of Cyclic Peptide Natural Products. *Future Med. Chem.* **2015**, *7* (16), 2121–2130.

(2) Lee, D.; Lee, S.; Choi, J.; Song, Y.-K.; Kim, M. J.; Shin, D.-S.; Bae, M. A.; Kim, Y.-C.; Park, C.-J.; Lee, K.-R.; Choi, J.-H.; Seo, J. Interplay Among Conformation, Intramolecular Hydrogen Bonds, and Chameleonicity in the Membrane Permeability and Cyclophilin a Binding of Macrocyclic Peptide Cyclosporin O Derivatives. *J. Med. Chem.* **2021**, *64* (12), 8272–8286.

(3) May, E. M. Lessons From Cyclosporine a: Structural Determinants of Conformation-Switching and Passive Membrane Penetration. *Doctoral Dissertation, Harvard University, Cambridge, MA,* **2018**, 1–145.

(4) Naylor, M. R.; Ly, A. M.; Handford, M. J.; Ramos, D. P.; Pye, C. R.; Furukawa, A.; Klein, V. G.; Noland, R. P.; Edmondson, Q.; Turmon, A. C.; Hewitt, W. M.; Schwochert, J.; Townsend, C. E.; Kelly, C. N.; Blanco, M.-J.; Lokey, R. S. Lipophilic Permeability Efficiency Reconciles the Opposing Roles of Lipophilicity in Membrane Permeability and Aqueous Solubility. *J. Med. Chem.* **2018**, *61* (24), 11169–11182.

(5) Sugita, M.; Sugiyama, S.; Fujie, T.; Yoshikawa, Y.; Yanagisawa, K.; Ohue, M.; Akiyama, Y. Large-Scale Membrane Permeability Prediction of Cyclic Peptides Crossing a Lipid Bilayer Based on Enhanced Sampling Molecular Dynamics Simulations. *J. Chem. Inf. Model.* **2021**, *61* (7), 3681–3695.
